# Effects of Relative Humidity on Dissolution and Carbonation Potential of Portlandite

**DOI:** 10.1021/acs.langmuir.5c05760

**Published:** 2026-02-23

**Authors:** Naohiko Saeki, Ippei Maruyama, Tulio Honorio

**Affiliations:** † Department of Architecture, Graduate School of Engineering, 13143 The University of Tokyo, Tokyo 113-8656, Japan; ‡ 12965 Université Paris-Saclay, CEA, Service de recherche en Corrosion et Comportement des Matériaux, Gif-sur-Yvette 91191, France

## Abstract

The carbonation process of cementitious materials is gaining attention as a way to mitigate anthropogenic CO_2_ emissions. Because the reactions occur as interfacial dissolution and precipitation within thin adsorbed water film on the substrate, understanding water adsorption and its influence on carbonation reactivity is essential. In this study, the water adsorption isotherms on portlandite surface were simulated by hybrid Grand Canonical Monte Carlo (GCMC)–Molecular Dynamics (MD) simulations and compared with the experiment. With the simulated water film under various relative humidity (RH), biased MD simulations were performed using a well-tempered metadynamics scheme to investigate how calcium dissolved from the portlandite surface. The results revealed that the dissolved Ca was likely to be trapped parallel to the substrate surface as inner-sphere adsorbed (adatom). The activation energies for detachment from the surface sites and for surface diffusion were calculated. In addition, the thermodynamical stability of the surface sites at various RH was evaluated by calculating the Gibbs energy for reaction (Δ_*r*_ *G*), showing a bilinear relationship with a decreasing trend until 40% RH and constant values above that, implying the threshold RH for Ca dissolution. Our simulations also revealed that the perpendicular movement of the dissolved Ca was restricted within where the H_2_O layer was present, which may spatially limit subsequent nucleation and crystal growth of calcium carbonate and inhibit complete carbonation of the substrate. For comparison with these simulations, experiments were also performed to study the degree of carbonation (DoC) of portlandite at various RH. The experimental trend showed good consistency with simulations with respect to the reactivity threshold and the reaction-saturated RH.

## Graphical abstract

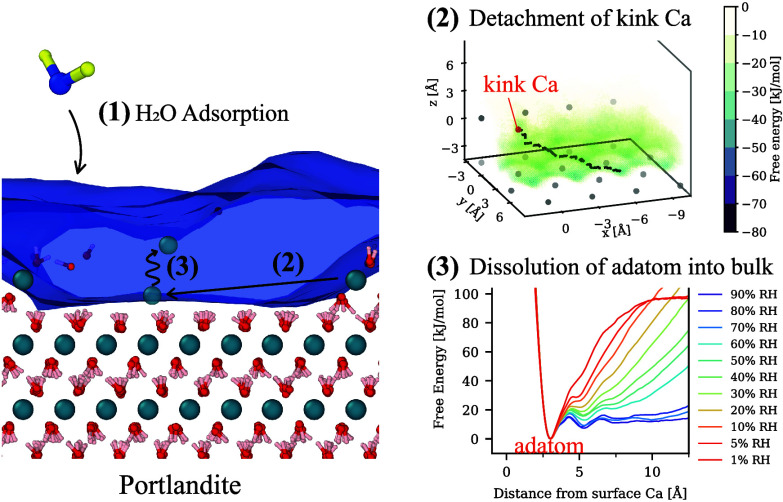

## Introduction

To mitigate global climate change in the Anthropocene, the ability of cementitious materials to chemically immobilize CO_2_ in a carbonation reaction has gained attention,^1–7^ during its curing period,^8,9^ service life,^10–13^ and after demolition,^14^ through which they can offset a fraction of CO_2_ emission originating from cement production. The most decisive factor in determining CO_2_ uptake is the water content, which is a function of external relative humidity (RH).^7,15–18^ On the one hand, the water content has an influence on the saturation degree of porous media.^19,20^ The proportion of gaseous and aqueous CO_2_ diffusion is thus governed by the water content, leading to the difference of the apparent diffusion coefficient as a function of RH.^21–24^ On the other hand, water works as a reaction medium because carbonation of cementitious materials is a dissolution–precipitation reaction^25,26^ (except the “dry carbonation” condition with *T* ≫ 100 °C^27^). The dissolution of solids, the dissolution of gaseous CO_2_, the speciation of aqueous CO_2_ for HCO_3_^–^ and CO_3_^2–^, and the nucleation and crystal growth of CaCO_3_ (Cc[Fn fn1])­require the presence of liquid water.

If the water content is too scarce, carbonation rarely occurs. This is known as the reactivity threshold.^15–18,28–32^ Various processes have recently been evoked to explain the origin of this threshold, including diffusivity,^33,34^ surface complex formation^35^ or activation energy for solid dissolution^36^ in a thin water film. Many studies showed that diffusion coefficients near the solid surface might be significantly smaller than those in bulk,^37–40^ but the effects may be surface dependent and need to also be evaluated for each mineral. Even when RH is higher than the threshold and carbonation is enabled, the degree of carbonation (DoC, the proportion of carbonatable calcium oxides converted to carbonates) does not always reach 100% at the intermediate RH.^15,16,18^ This is generally understood as the formation of Cc that acts as a protective layer for the substrate, which forms within the thin water film and adheres to the substrate surface.^41–43^ When RH increases further, the protective Cc layer becomes more permeable and the carbonation of the substrate continues successively, DoC reaching ∼100%.

In this way, the thin water film adsorbed on the surface governs the overall carbonation reactivity. Given that, this study aims to elucidate the influence of adsorbed water film on carbonation. We investigated portlandite (Ca­(OH)_2_, or CH in the nomenclature of cement chemistry), which accounts for ∼20% mass of the hydrated cement paste^44^ and is the main source of calcium available for carbonation (although other hydrate phases such as C–S–H, ettringite, layered double hydroxide, or hydrogarnet also contribute to carbonation, CH reacts most rapidly among them^18^).

In the first part of this article, to study the amount and properties of adsorbed H_2_O at a given RH, a hybrid simulation was performed combining Grand Canonical Monte Carlo (GCMC) and Molecular Dynamics (MD) and compared with experimental sorption isotherms. GCMC-MD simulation is a well-established method and has been used for the adsorption studies on silica,^45,46^ C–S–H (poor-crystalline calcium silicate hydrate),^47^ and various minerals.^48,49^

In the second part, dissolution of CH and its relevant thermodynamic properties were simulated with a rare event sampling method using metadynamics.^50,51^ This method is suitable to overcome an inherent problem of classical MD simulation, that the system might remain trapped in the local minima basin within the time scale accessible to standard MD, by adding biased potentials along the predefined collective variables (CV). Energy barrier calculations for the dissolution of cementitious minerals have already been performed,^52–55^ and some studies successfully scaled the activation energies obtained to macroscopic kinetics using a Kinetic Monte Carlo method.^56,57^ Although these simulations could reproduce the dissolution behavior, the effect of the thin adsorbed water film has been rarely investigated. Using the water films obtained in the first part, we investigated how RH affects the dissolution progress. The results were discussed in comparison of experiments, in which carbonation reactivity of CH was measured at various RH.

## Methodology

### Water Adsorption Experiment

Water vapor adsorption experiment was performed with BELSORP MAX X (Microtrac BEL). One gram of CH reagent powder with purity 99.9% (FUJIFILM Wako) was predried prior to the analysis under vacuum (FLOVAC Degasser, Quantachrome, with the absolute pressure reached during this experiment being ∼ 5 Pa) at 105 °C for 2 h. Adsorption isotherms were measured at 20 °C for three samples for repeatability, with a pressure tolerance of 0.3% of variation during 5 min. BET theory was used with RH range of 5 to 35% to calculate the surface area.^58^ The effective cross-sectional area of H_2_O was 0.114 nm^2^ per H_2_O molucule.^59^

### Water Adsorption Simulation

The crystal structure of CH for the simulations was taken from Materials Project^60^ (number mp-23879). The unit cell size is *a* = *b* = 3.58 and *c* = 4.82 Å with α = β = 90° and γ = 120°. The morphology of CH was previously studied,^61–64^ which showed that a hexagonal platelet CH was often observed in the hydrated cement paste. As shown in Figure 1, {1 0 0} surface includes all the six equivalent side planes of a hexagonal portlandite crystal, so (0 1 0) surface was investigated as a representative of {1 0 0} in this study.

**Figure 1:**
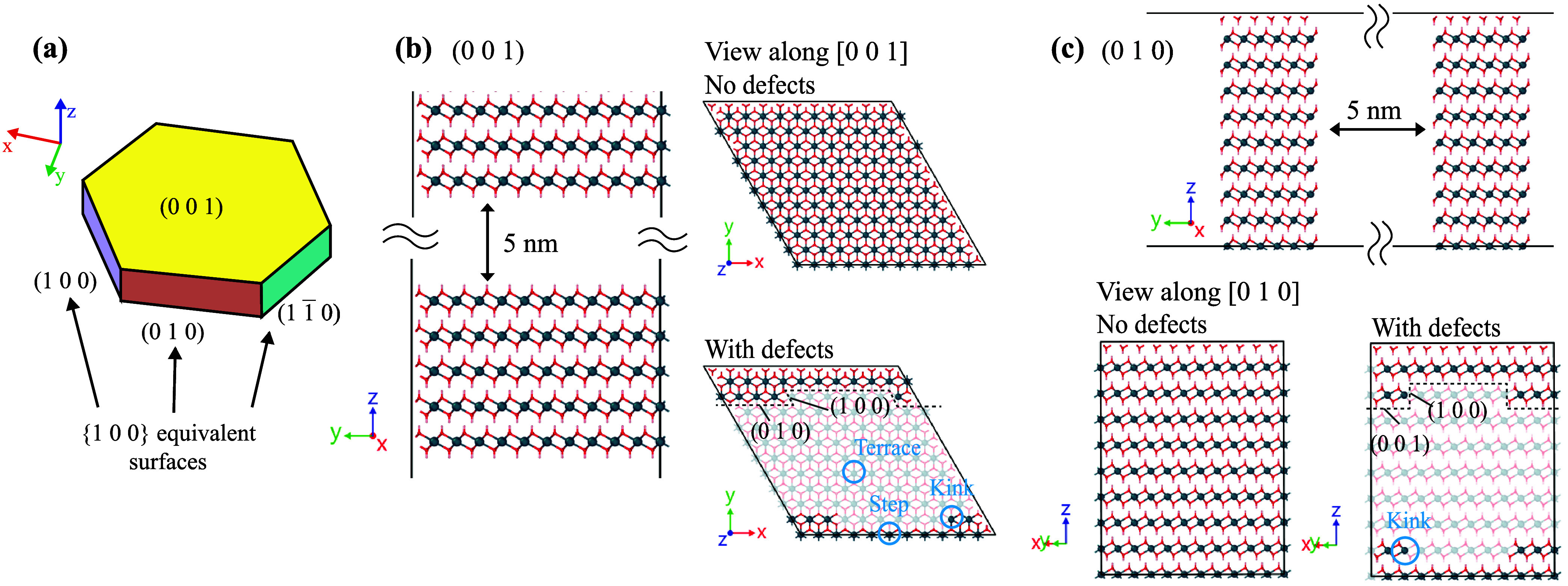
(a) Illustration of hexagonal CH plate. (b) investigated surfaces with/without surface defects for (0 0 1) and (c) for (0 1 0). Blue = calcium, red = oxygen, and pink = hydrogen. The translucent layers in surfaces with defects show monolayer-depth etch pits.

We replicated the unit cell to create a surface area of 21.48 × 21.70 Å^2^ for (0 0 1) and 21.48 × 24.11 Å^2^ for (0 1 0). Each structure was also duplicated perpendicular to their surfaces at least 20 Å (five or six layers). Then, a 5 nm vacant space was inserted between the cleaved surfaces (Figure 1b,c). (0 0 1) had a perfect cleavage plane, and its surface terminated with both planes exposing OH^–^. In contrast, creating cleaved planes of (0 1 0) required removals of one surface OH^–^ per Ca to maintain whole charge neutrality. As a result, the coordination number of surface Ca decreased from six to five. Next, to investigate the effects of surface defects, monolayer-depth etch pits were created as shown in Figure 1. Different surface sites were created, e.g., terrace, step, and kink.^65,66^

All simulations were performed with LAMMPS.^67^ The periodic boundary condition was applied for all dimensions. ClayFF was used^69,70^ with the rigid SPC/E water model.^71^ This is one of the most successful force fields to simulate interfacial properties among various ones (see the review of Mishra et al.^72^ for comparison). The parameters used are summarized in the Supporting Information. Long-range electrostatic interactions were handled using the particle–particle particle–mesh (PPPM) method with an accuracy of 10^–5^. A cutoff distance of 9 Å was adopted for Lennard–Jones interactions and tail corrections were applied. The simulation time step was set to 1 fs.

A hybrid GCMC-MD simulation was performed in which Monte Carlo insertions and deletions of H_2_O were performed in the grand canonical ensemble (μVT) and MD simulations in the canonical ensemble (NVT). In GCMC, the number of H_2_O molecules changed with time according to the chemical potential imposed by an imaginary external reservoir, while the numbers of Ca and OH^–^ were fixed.^73^ The pressure corresponding to various RH [%] is defined using *P*_res_ = RH *P*_sat_, where *P*_sat_ is the saturated vapor pressure. At a given temperature *T*, *P*_sat_ is estimated by the Clausius–Clapeyon equation:

1 $$P_{sat}\left(T\right)=P_{ref}\;\exp\left[\frac{\Delta H^{vap}}{R}\left(\frac{1}{T_{ref}}-\frac{1}{T}\right)\right]$$

where *P*_ref_ is the saturated vapor pressure at the reference temperature *T*_ref_. We used the value in NIST standard^74^ of *P*_ref_ = 0.01004 atm at *T*_ref_ = 300 K for bulk SPC/E water. Δ*H* ^vap^ is the apparent enthalpy of vaporization. Following the results of Fugel et al.,^75^ Δ*H* ^vap^ is independent of temperature and is assumed to be 44.2 kJ/mol for SPC/E water. *R* is the gas constant. It is noted that the saturation pressure of the water model in MD slightly deviates from the experimental one;^76^ nevertheless, a previous sorption simulations with SPC/E water under eq 1 successfully reproduced the adsorption/desorption behavior of cementitious materials.^77^

At the beginning of the simulations, a small number of H_2_O molecules (six) were manually introduced above the cleaved surfaces, so that the system could be initialized with a nonzero number of water molecules (this was done because having zero water molecules can cause issues in the calculation of probabilities in GCMC, which involves division by the number of exchanging species during insertion/deletion Monte Carlo moves). MD simulations were performed in the NVT ensemble with the Nosé–Hoover thermostat with the temperature damping factor being 100 fs,^78,79^ and in every 100 MD steps, 1000 GCMC trials were attempted to insert or remove H_2_O molecules. It is noted that translation and rotation of H_2_O were not attempted in GCMC movements because they could be supplemented by MD runs. One nanosecond simulation time (corresponding to 1 million MD steps and 10 million GCMC trials) were performed to equilibrate the number of H_2_O (equilibration run, see Supporting Information for the evolution of the number). Subsequently, another 2 million GCMC trials were carried out (production run) to obtain the average and standard deviation over the relevant observables. Three simulations were run independently to take the average.

### Simulations on the Property of Adsorbed Water

During the production run, the density profile of adsorbed H_2_O perpendicular to the surfaces and the average coordination number of surface Ca to Oh (hydroxyl oxygen) and Ow (water oxygen) atoms were also obtained. In addition, the isosteric enthalpy of adsorption (Δ*H* ^ads^) was calculated using the fluctuation formula.^80–82^

2 $$\Delta H^{\mathrm{ads}}=\frac{\left\langle \mathrm{UN}\right\rangle -\left\langle U\right\rangle \left\langle N\right\rangle }{\langle N^{2}\rangle -\langle N\rangle^{2}}-\mathrm{RT}$$

where *U* and *N* are the potential energy and number of H_2_O, respectively. ⟨*A*⟩ is the time-average of some property *A* during the production run. The denominator ⟨*N* ^2^⟩–⟨*N*⟩^2^ represents the variance of *N* and the numerator ⟨*UN*⟩–⟨*U*⟩⟨*N*⟩ represents the covariance between *U* and *N*.

The self-diffusion coefficient of adsorbed H_2_O was also calculated. Since the number of H_2_O must be constant for this calculation, NVT runs without GCMC trials were applied starting from the atomic configurations after equilibration runs. Ten nanosecond simulation was performed. The lateral diffusion to the surface, *D*_∥_, was calculated using the Einstein formula.

3 $$D_{∥}=\frac{1}{4}\lim_{t\to \infty }\frac{\mathrm{MS}D_{x}+\mathrm{MS}D_{y}}{t}$$

where *MSD*_*i*_ (*i* = *x*, *y*) is the mean square displacement (*x* and *y* are two orthogonal directions parallel to the surface). The perpendicular diffusion to the surface, *D*_⊥_, confined within the thickness of the water film, was calculated using a relationship proposed by Kusumi et al.,^83^

4 $$\mathrm{MS}D_{z}\left(t\right)=\frac{H^{2}}{6}-\frac{16H^{2}}{\pi^{4}}\sum_{n=1\left(odd\right)}^{\infty }\frac{1}{n^{4}}\;\exp\left[-D_{⊥}{\left(\frac{n\pi }{H}\right)}^{2}t\right]$$

where *MSD*_*z*_ (*t*) is the mean square displacement perpendicular to the surface and *H* is the confinement thickness. At *t* → ∞, *MSD*_*z*_ (*t*) approaches asymptotically to $\frac{H^{2}}{6}$ . This confinement thickness has been generally taken as the pore thickness in the study of nanoporous cement system.^40^ Here, *H* should be understood as the effective thickness of the adsorbed water layer confined between the CH surface and the interface with the empty pore. In other words, *MSD*_*z*_(*t*) evaluated at a sufficiently large time scale informs on the thickness of the adsorbed H_2_O layer. The perpendicular diffusion coefficient *D*_⊥_ can be obtained by fitting the *MSD*_*z*_ (*t*)—*t* relationship to eq 4. We fitted *D*_⊥_ with the least-square method using the initial 5 ps simulation for (0 0 1) and 20 ps for (0 1 0). The time ranges used for the calculations were determined where the linear relationship between MSD and *t* was maintained.

### Carbonation Experiment

The same CH reagent powder was used as in the adsorption measurements to test the carbonation reactivity under different RH. CH was placed in a handmade carbonation system described in our earlier studies,^17^ where air pumps constantly supplied RH-controlled air to carbonation desiccators. RH was controlled with saturated salt solutions^84^ with KNO_3_ (equilibrium RH at 20 °C is 95%), (NH_4_)_2_SO_4_ (81%), NaCl (75%), KI (69%), NaBr (59%), K_2_CO_3_ (43%), MgCl_2_ (33%), CH_3_COOK (23%), and LiCl (11%). The RH in the desiccators during carbonation was monitored with data loggers (TR-76Ui, T&D) and are shown in the Supporting Information.

The degree of carbonation (DoC) was measured at 0, 3, 7, 28, and 56 days of carbonation with thermogravimetry (STA 2500, Netzsch). Twenty milligrams of samples were heated from room temperature to 900 °C at a rate of 20 °C/min. 70 mL/min of N_2_ flow was purged. The weight loss from the peaks of CH and Cc were integrated with the tangential method to estimate their amount.^85,86^ Then, DoC was calculated either by (i) the remaining amount of CH or (ii) the precipitated amount of Cc. For the former,

5 $$\mathrm{Do}C_{t,\mathrm{Ca}{\left(\mathrm{OH}\right)}_{2}}=1-\frac{m_{\mathrm{CaO}}}{m_{\mathrm{Ca}{\left(\mathrm{OH}\right)}_{2}}}M_{t,\mathrm{Ca}{\left(\mathrm{OH}\right)}_{2}}$$

For the latter,

6 $$\mathrm{Do}C_{t,{\mathrm{CaCO}}_{3}}=\frac{m_{\mathrm{CaO}}}{m_{\mathrm{CaC}O_{3}}}M_{t,{\mathrm{CaCO}}_{3}}$$

where *M*_*t*, Ca(OH)_2__ and *M*_*t*,CaCO_3__ are the mass of CH and Cc per gram of ignited CH [g/g-CaO] after carbonation for *t* days. *m*_CaO_, *m*_Ca(OH)_2__, and *m*_CaCO_3__ are the molar masses, which are 56.08, 74.09, and 100.09 [g/mol], respectively.

### Calcium Dissolution Simulation

Biased MD simulations were performed with LAMMPS patched with PLUMED software^87^ to study the dissolution of CH. CH surfaces (with defects) after the GCMC-MD equilibration runs were used as initial configurations. Since there is a dependency of the free energy surface (FES) on different surface sites,^54,56^ Ca at a kink position of (0 0 1) and (0 1 0), and at a step and a terrace positions were considered in this study. The positions of the dissolved Ca corresponded to the blue circles in Figure 1.

A well-tempered metadynamics scheme was applied to explore the FES.^50,51^ In well-tempered metadynamics, Gaussian potential(s) are constantly added on-the-fly during molecular dynamics. The sum of potentials, *V*(***s***,*t*′), is described as^88^

7 $$V\left(s,t^{\prime }\right)=\sum_{i}\sum_{t<t^{\prime }\;}W\exp\left(-\frac{V\left(s\left(t\right)\right)}{\Delta T}\right)\exp\left(-\frac{{\left(s_{i}\left(t\right)-s_{i}\right)}^{2}}{2\sigma_{i}^{2}}\right)$$

where *t*′ is the current time, ***s*** = (*s*_1_, ..., *s*_*i*_, ...) is a set of collective variables (CV), *W* is the initial Gaussian height, Δ*T* is a parameter that controls the Gaussian height, and σ is the Gaussian width. After convergence, the FES along selected CVs is equal to the negative of *V*(***s***, *t*′).

The selection of appropriate CVs is nontrivial. Previous studies used a variety of CVs for dissolution simulation: distance between the dissolved atom and the center of mass of the entire crystal,^54^ perpendicular displacement of the dissolved atom,^55^ a combination of perpendicular displacement and coordination number with Ow atoms,^36^ to name a few. We selected *x*, *y*, and *z* displacements of the dissolved Ca from its original position as three individual CVs because it could capture the whole movement of the dissolved atom in Cartesian coordinates and help identify multiple stable configurations.^52,56,89^ The Gaussian potentials for each CV were set to 0.1 Å, and the height was 0.3 kcal/mol (=1.25 kJ/mol). They were added every 100 MD time step. The bias factor was set to 20. The simulations were performed for 40 ns and 400,000 Gaussian biases were accumulated in total.

The minimum energy paths (MEP) at each RH and the free energies along them were extracted with the MULE software that implemented Dijkstra’s algorithm.^90^ As discussed later, the FES with the three Cartesian CVs could capture the detachment of Ca from the surface sites to the adatom (adsorbed atom) sites and the lateral transitions between the adatoms. However, exploring FES when the Ca moved into the bulk solution was extremely time-consuming and computationally prohibitive.^56^ Therefore, we also launched another metadynamics simulation with a reduced number of CVs. In this simulation, the atomic configurations where the dissolved Ca was at the adatom position were set as starting points, and then the perpendicular displacement to the surface was set as a single CV. The parameters for the Gaussian potential and the simulation time were the same as in the first simulation.

We used ClayFF for the dissolution simulation. The choice was, for one thing, to carry out end-to-end simulations with the GCMC-MD adsorption simulation. Another reason was the simplicity and low computational cost of ClayFF. For this reason, ClayFF and relevant nonreactive force fields have been applied in many previous metadynamics studies.^52,91,92^ Some have utilized reactive force fields such as ReaxFF^93^ in a metadynamics scheme, but their investigations were only one- or two-dimensional.^35,94^ The exploration of three-dimensional CVs in this study practically inhibited the application of ReaxFF for a large number of simulations. According to our preliminary test with the ReaxFF parameters (Supporting Information),^95–97^ even 4 ns of simulation, which took more than 1.5 months using 48 cores MPI parallelization, was not enough to explore the entire FES in the Cartesian space.

The composition of the solution should be mentioned. The solubility of CH is 0.012 [mol/L],^98^ which corresponds to one mol of dissolved Ca­(OH)_2_ in 4630 mol of H_2_O (the deviation of the solubility is described in detail in the Supporting Information). In our simulation, even the highest RH (90%) had ∼ 500 H_2_O molecules on the adsorbed surface. Therefore, once a single Ca­(OH)_2_ cluster dissolved, it immediately caused a huge supersaturation. Another issue was that the dissolution simulations were performed without the coexistence of other ion species in the solution. In carbonation, carbonate ion species are present. Hydrated cementitious pastes also contain multiple ion species such as alkali ions (sodium, potassium) and those dissolved from the clinker (silica, aluminum and iron-related species).^99,100^ This would introduce another equilibrium relationship other than the C–Ca–O–H systems.

## Results and Discussion

### Comparison of Water Adsorption between Experiments and GCMC-MD Simulation

The experimental isotherm at 20 °C and the simulated ones for (0 0 1) and (0 1 0) without defects are summarized in Figure 2a. The raw experimental isotherms and BET analysis are shown in the Supporting Information. The adsorbed amount was divided by its BET surface area, then the unit was converted to [g/m^2^] for comparison with simulations. The shape of the isotherm corresponded to the IUPAC type II classification.^101,102^ A strong adsorbent–adsorbate interaction resulted in a sharp knee at low RH. A linear range started from ∼10% RH, which is generally explained in association with the completion of monolayer coverage. This linear relationship continued until ∼60% RH, creating multilayer adsorption. Above that RH, the slope increased steeply again, possibly due to capillary condensation. The shape of the experimental isotherm agreed well with previous studies.^41,103^

**Figure 2:**
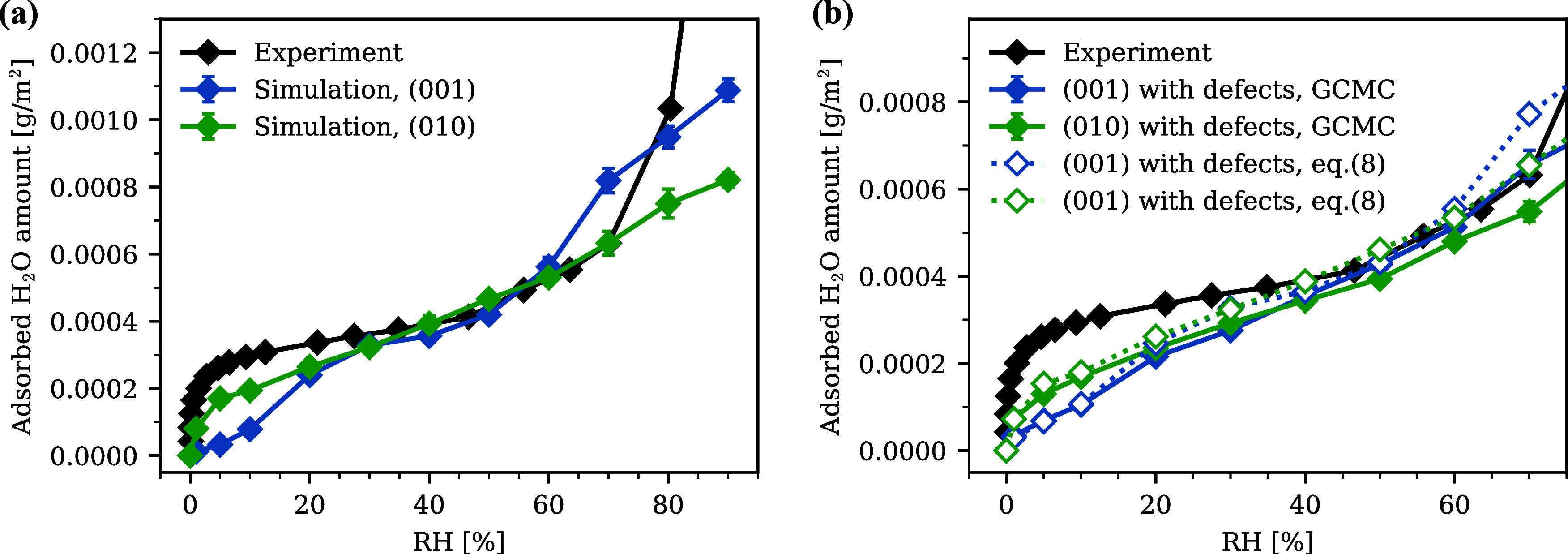
(a) The experimental isotherm at 20 °C and simulated isotherms for (0 0 1) and (0 1 0) surfaces without defects. (b) The simulated isotherms for (0 0 1) and (0 1 0) with surface defects (denoted as GCMC) and the estimated isotherms using eq 8.

The simulated isotherms showed a difference in shape between the two surfaces. The adsorbed H_2_O on (0 0 1) was very limited below 20% RH. This low-RH shape corresponded to the IUPAC type III or V, which is attributed to relatively weak adsorbent–adsorbate interactions.^101,102^ In contrast, a sharp increase was observed at low RH on (0 1 0), which produced a shape similar to that of the experiment. As RH increased, the adsorption from the two surfaces took a similar value at 20% RH, and their isotherms almost overlapped in the linear range at 20–60% RH.

The simulated isotherms for surfaces with defects are shown in the solid lines in Figure 2b. For comparison, we also estimated the adsorption on these defected surfaces using isotherms of defect-free surfaces. As shown in Figure 1b, (0 0 1) surface with defects had etch pits along [0 1 0] and [1 0 0] directions. Therefore, adsorption can be schematically divided into that from the (0 0 1) terrace surface and that from the monolayer-depth {0 1 0} surface. Taking the weighted average, adsorption on the defected surfaces per surface area was estimated by eq 8.

8 $$W_{001,\mathrm{defect}}=\frac{W_{001}A_{001}+W_{010}A_{010}}{A_{001}+A_{010}}$$

where *W*_001_ and *W*_010_ are the adsorption on (0 0 1) and (0 1 0) defect-free surfaces [g/m^2^] and *A*_001_ and *A*_010_ are the proportion of each surface area to the overall surface area (*A*_001_ + *A*_010_ = 1). Adsorption on (0 1 0) with defects, *W*_010,defect_, was estimated in the same manner. From the geometry in Figure 1, (*A*_001_, *A*_010_) = (0.75, 0.25) for the (0 0 1) surface with defects and (0.12, 0.88) for (0 1 0).

Figure 2 shows that the GCMC results and the estimations from eq 8 lie on almost the same curves. This means that the adsorption isotherms on defected surfaces can be well estimated from the weighted average of the defect-free surfaces.

### Simulated Properties of Adsorbed Water

The isosteric enthalpy of adsorption, Δ*H* ^ads^, on (0 0 1) and (0 1 0) is shown in Figure 3. Δ*H* ^ads^ was governed by the adsorbent–adsorbate interaction at lower RH, and as RH increased, Δ*H* ^ads^ increased and approached the bulk enthalpy of vaporization (−44.2 kJ/mol from a benchmark calculation using bulk SPC/E water^75^) as previously shown.^45,46^ When comparing (0 0 1) and (0 1 0), the latter surface tended to take lower Δ*H* ^ads^ values than the former, although the differences were within the uncertainty/variability of the simulations. This suggests that the adsorbed H_2_O might be more strongly attracted to the substrate CH in the latter case, but further simulations with more precise results would be needed to confirm this aspect.

**Figure 3:**
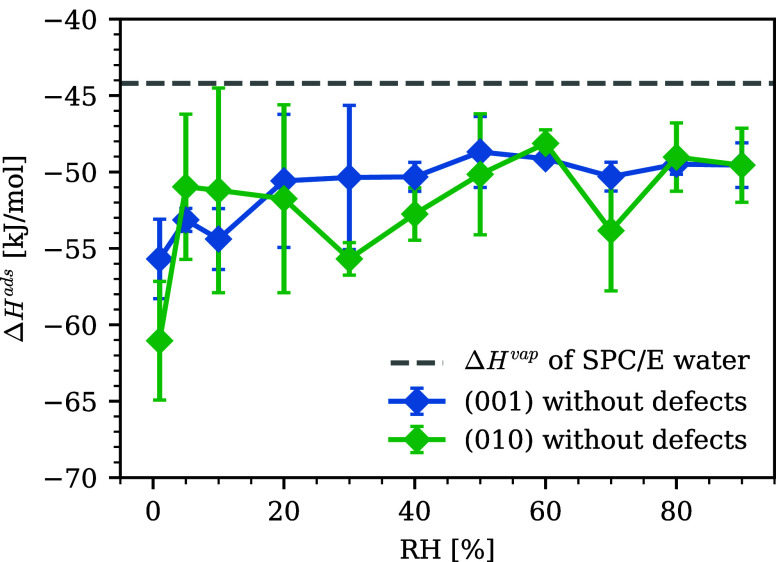
Isosteric enthalpy of adsorption for the (0 0 1) and (0 1 0) surfaces without defects. The bulk enthalpy of vaporization for SPC/E water was taken from ref 75.

The possible stronger adsorbent–adsorbate interaction on the (0 1 0) surface could be attributed to the positions of the adsorbed H_2_O. In Figure 4, snapshots of the adsorbed H_2_O (equilibrated at 20% RH) and density profiles of Ow atoms in H_2_O perpendicular to the surfaces at various RH are summarized. The *x*-axis of the density profile is the relative distance between Ow atoms in H_2_O and Ca atoms on the surface. As snapshots and the average coordination number (cn) of Ca on the surface show, all Ca atoms on (0 0 1) were fully covered by OH^–^ groups and 6-fold coordinated only with Oh atoms. The monolayer peak (*W*_1_) formed at >3 Å away from Ca, which was longer than the closest Ca–O distance,^104,105^ meaning that adsorbed H_2_O was not directly bonded to Ca. This “prewetted” configuration^45^ was the cause of the weak interaction between Ca and Ow, which resulted in little H_2_O adsorption below 10% RH (Figure 2a). In contrast, Ca atoms on (0 1 0) were only 5-fold coordinated with the Oh atoms, and the other bond was used to connect to the adsorbed Ow atoms.^35^ This resulted in a strong attraction between Ca and Ow, which allowed the adsorption even at very low RH (Figure 2).

**Figure 4:**
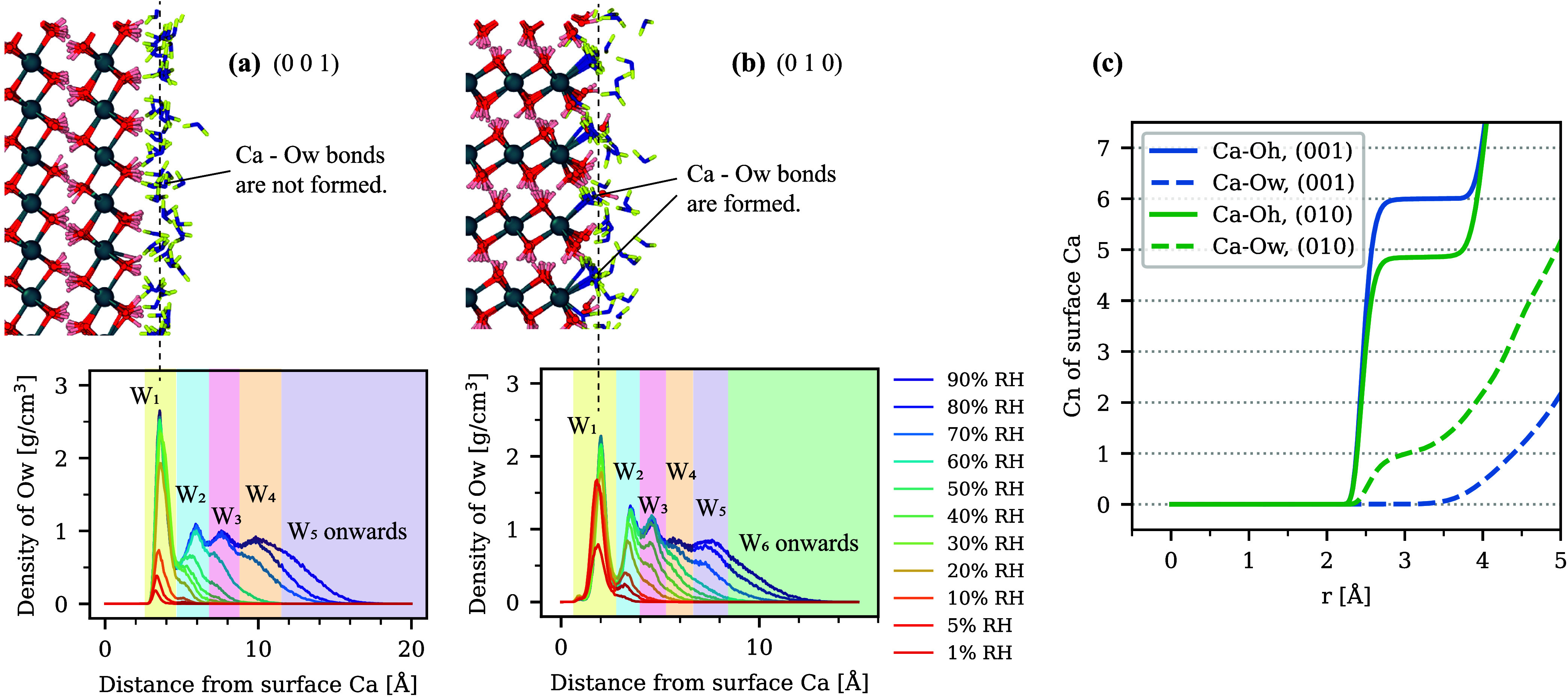
(a) A snapshot equilibrated at 20% RH (top) and density profiles of Ow atoms in H_2_O perpendicular to the surface at various RH (bottom) for the (0 0 1) and (b) (0 1 0) surfaces. The colors of atoms in snapshots are the same as in Figure 1, with blue-yellow bars being H_2_O molecules. (c) Averaged coordination number (cn) of surface Ca with Oh and Ow atoms.

The average MSD and self-diffusion coefficient of adsorbed H_2_O are summarized in Figure 5. The self-diffusion coefficient of the bulk SPC/E water at 300 K was taken from a previous study.^40^ For both (0 0 1) and (0 1 0), MSD_∥_ increased linearly with time, which was a hallmark of normal/Fickean diffusion. Overall, MSD_∥_ increased monotonically with increasing RH. The exception occurred at very low RH, where too small number of H_2_O in the system made the MSD more susceptible to be affected by molecules occasionally detaching from the adsorbed water layer (a faster displacement in the vapor state led to large average MSD). With a sufficient number of the adsorbed H_2_O molecules with respect to vaporized ones, the increase in MSD_∥_ with increasing RH was intuitive because the adsorbate–adsorbent interaction decreased with a larger distance from the adsorbent surface, which led overall to a less constrained environment for a larger portion of adsorbed H_2_O at higher RH. MSD_⊥_ showed a sharp increase until hundreds of ps and asymptotically approached constant values after that, which is typical for diffusion in confined space.^40,83^ The horizontal asymptotes were a function of the effective water film thickness *H*. Using eq 4, *H* was calculated and summarized in Table 1. MSD_⊥_ and *H* monotonically increased with an increase in RH.

**Figure 5:**
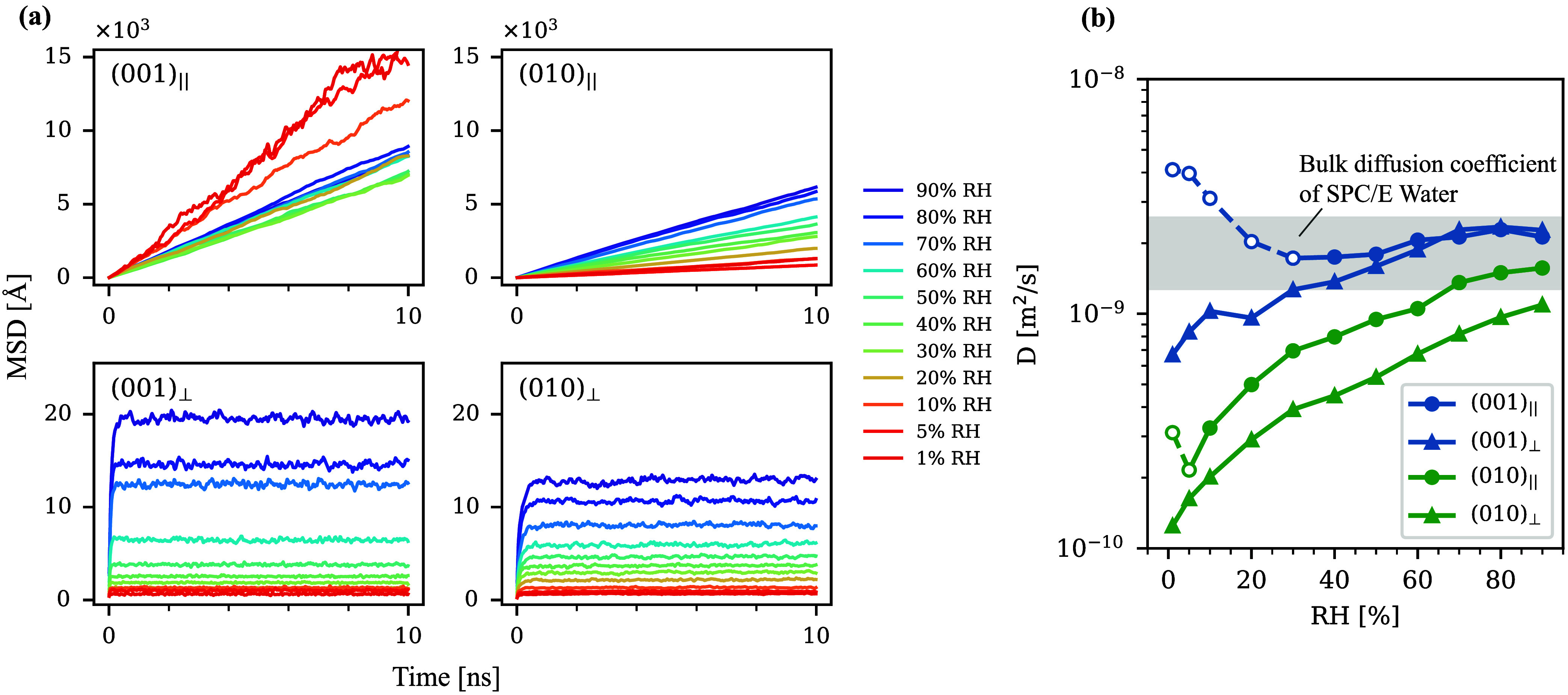
(a) MSD parallel (MSD_∥_) and perpendicular to the surface (MSD_⊥_). MSD are the average of three independent simulations. (b) Parallel and perpendicular self-diffusion coefficients of adsorbed H_2_O. Bulk diffusion coefficient of SPC/E water at 300 K was taken from Honorio et al.^40^

**Table 1:** Effective Water Film Thickness on Defect-Free (0 0 1) and (0 1 0) Surfaces in Å, Calculated from the Horizontal Asymptotes in Figure 5 and eq 4

	RH (%)										
	1	5	10	20	30	40	50	60	70	80	90
(0 0 1)	1.99	2.48	2.79	2.79	3.34	3.91	4.78	6.22	8.61	9.37	10.83
(0 1 0)	2.04	2.35	2.82	3.68	4.26	4.73	5.30	6.06	6.92	8.01	8.85

From the density profile of H_2_O, the average number of H_2_O molecules in each water layer (colored in Figure 4) per surface area of CH was extracted and plotted in Figure 6. It shows that the formation of the monolayer peak (*W*_1_) completed at 30% RH for (0 0 1), while it completed at 5% RH for (0 1 0). However, the adsorbed H_2_O for (0 1 0) at such a low RH was more strongly attracted to the adsorbent, so it was less mobile. Therefore, when comparing the self-diffusion coefficient between (0 0 1) and (0 1 0), the former had higher values.

**Figure 6:**
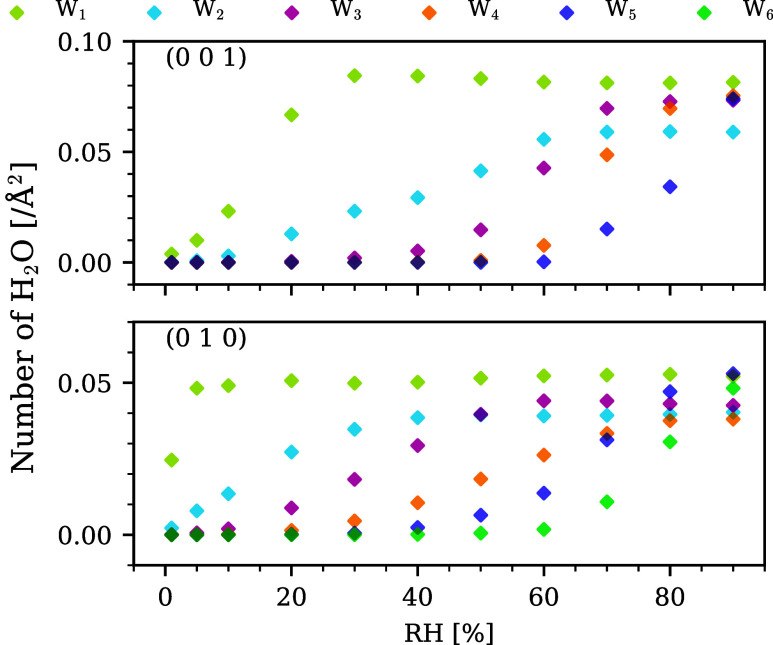
Average number of H_2_O molecules per surface area of CH on (0 0 1) (top) and (0 1 0) (bottom) in each water layer.

### Experimental Carbonation Reactivity

DTG (derivative TG) curves from samples at different carbonation periods and RH are summarized in Figure 7. The peak of CH dehydration appeared around 350 to 540 °C, which decreased as carbonation progressed. Meanwhile, the Cc decomposition peak around 540 to 850 °C increased. Using eqs 5 and 6, DoC was calculated in Figure 8. DoC from the remaining amount of CH was slightly higher than that from the precipitated amount of Cc, which may be derived from the formation of amorphous (or less crystalline) Cc that decomposes at lower temperature than 500 °C.^106–108^ It is noted that data at 56 d at 59 to 95% RH were not measured because DoC reached stable values after 7 d or the remaining amount of CH became almost zero.

**Figure 7:**
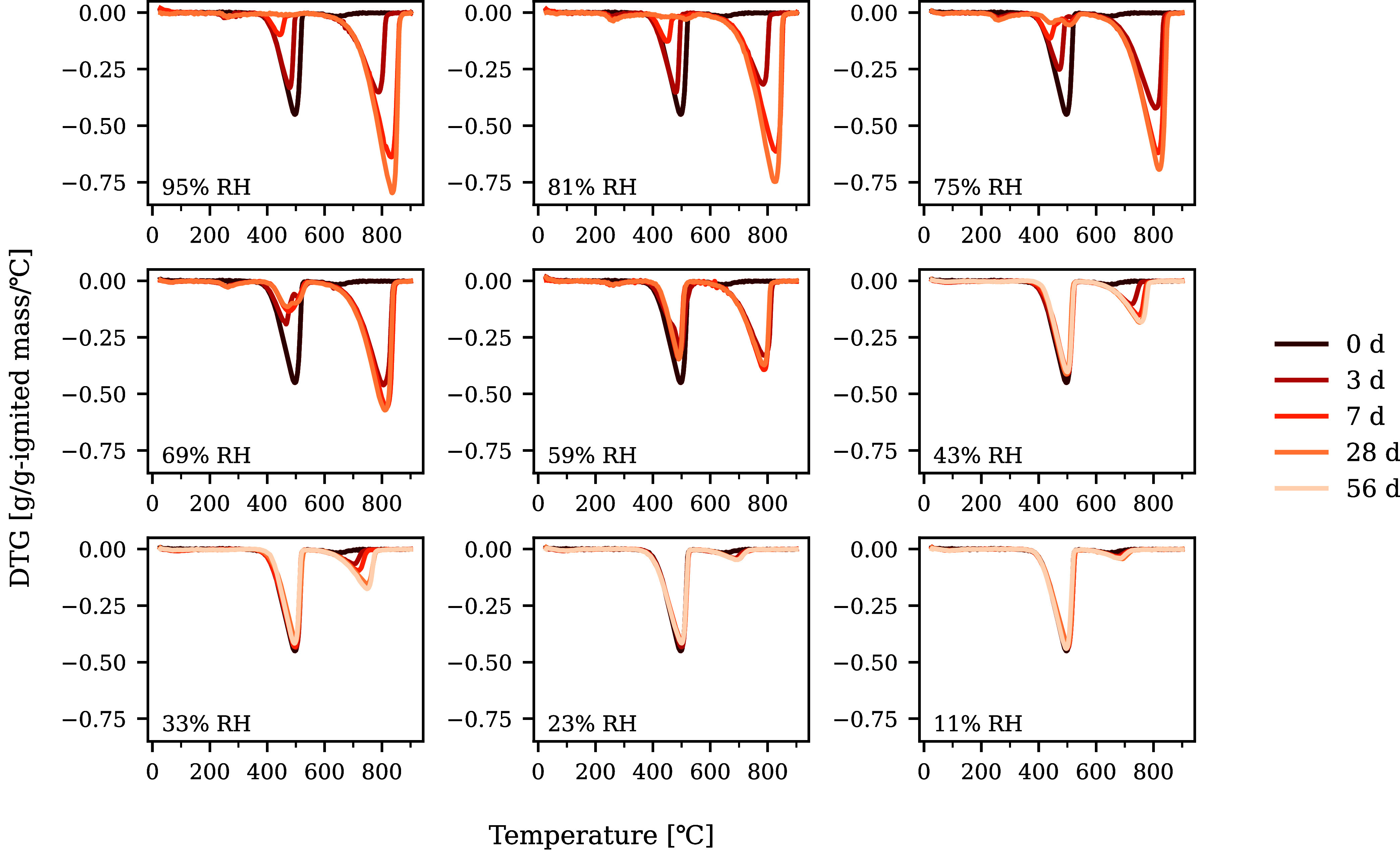
Derivative TG curves of carbonated CH samples at different RH.

**Figure 8:**
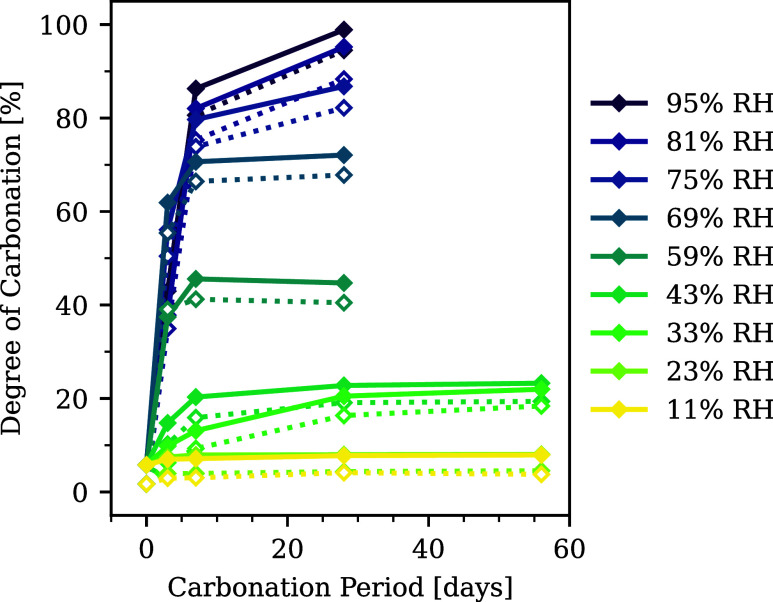
Degree of carbonation (DoC) of carbonated CH at different RH. The solid lines show DoC_*t*,Ca(OH)_2__ calculated from eq 5. The dotted lines of the same colors show DoC_*t*,CaCO_3__ from eq 6.

At 81% RH or above, DoC quickly increased in the initial carbonation period and reached ∼100% at 28 d. The completion of dissolution at these high RH was also observed in our previous studies.^17,18^ At 69 and 59% RH, the initial reaction rate corresponded to those at higher RH, but the slope gradually loosened. DoC reached a plateau after 7 d of carbonation. The slowdown or even stoppage of carbonation at the intermediate RH is consistent with many previous experiments.^15–17^ At 43 and 33% RH, carbonation still occurred, but the initial reaction rate and the final DoC values after 56 d were much lower than at higher RH.

At 23% RH or below, DoC rarely increased from the beginning, suggesting that the threshold RH that allowed CH carbonation lay between 23 and 33%. This conclusion is consistent with the observation of Dheilly et al.^31^ The threshold RH was reported to be ∼10% at higher temperature.^30^ In fact, this threshold does not depend on the presence of CO_2_; Yang et al. performed in situ atomic force microscopy (AFM) observations under CO_2_ free atmosphere and also concluded that CH dissolution was enabled around 30% RH.^32^ The similar threshold RH between carbonation and dissolution can imply that the dissolution behavior is a key factor to determine the overall carbonation reactivity.

### Simulated Dissolution of Portlandite

Free energy surfaces (FES) of Ca dissolution from the (0 0 1) kink site at 10, 40, and 70% RH are plotted in Figure 9, with the minimum energy paths (MEP) overlaid. The results on other RH are summarized in the Supporting Information. At all RH, hexagonal FES patterns parallel to (0 0 1) were observed, which described lower free energy sites in Cartesian coordinates (i.e., where the dissolved Ca ion was more likely to stay). These hexagons appeared at a position of ∼1.6 Å lower *z* position than the original kink site. According to the MEP, the dissolved Ca first moved to a lower *z*-coordinate position and then followed a path along the hexagonal pattern. The local minimum energy sites were located at the vertices of the hexagons, named S_2_ to S_4_ here, above the gravimetric center of their surrounding three Oh atoms (see the snapshots). The distance of Ca–Oh was ∼2.6 Å, which was a typical bond length. Although there was electrostatic repulsion between Ca and Hh (hydroxyl hydrogen), surface Hh atoms bent outward from the Ca and the repulsion lessened. Rather, a stronger attraction between Ca and Oh (the effective charge of Oh doubles that of Hh) made these sites energetically favorable.^109^ These sites were inner-sphere (IS) adsorbed, that is, having a direct chemical bond to the substrate surface. It is noted that this hexagonal pattern was also observed with the simulation using ReaxFF parameters (Supporting Information), suggesting that the FES did not depend on the force field.

**Figure 9:**
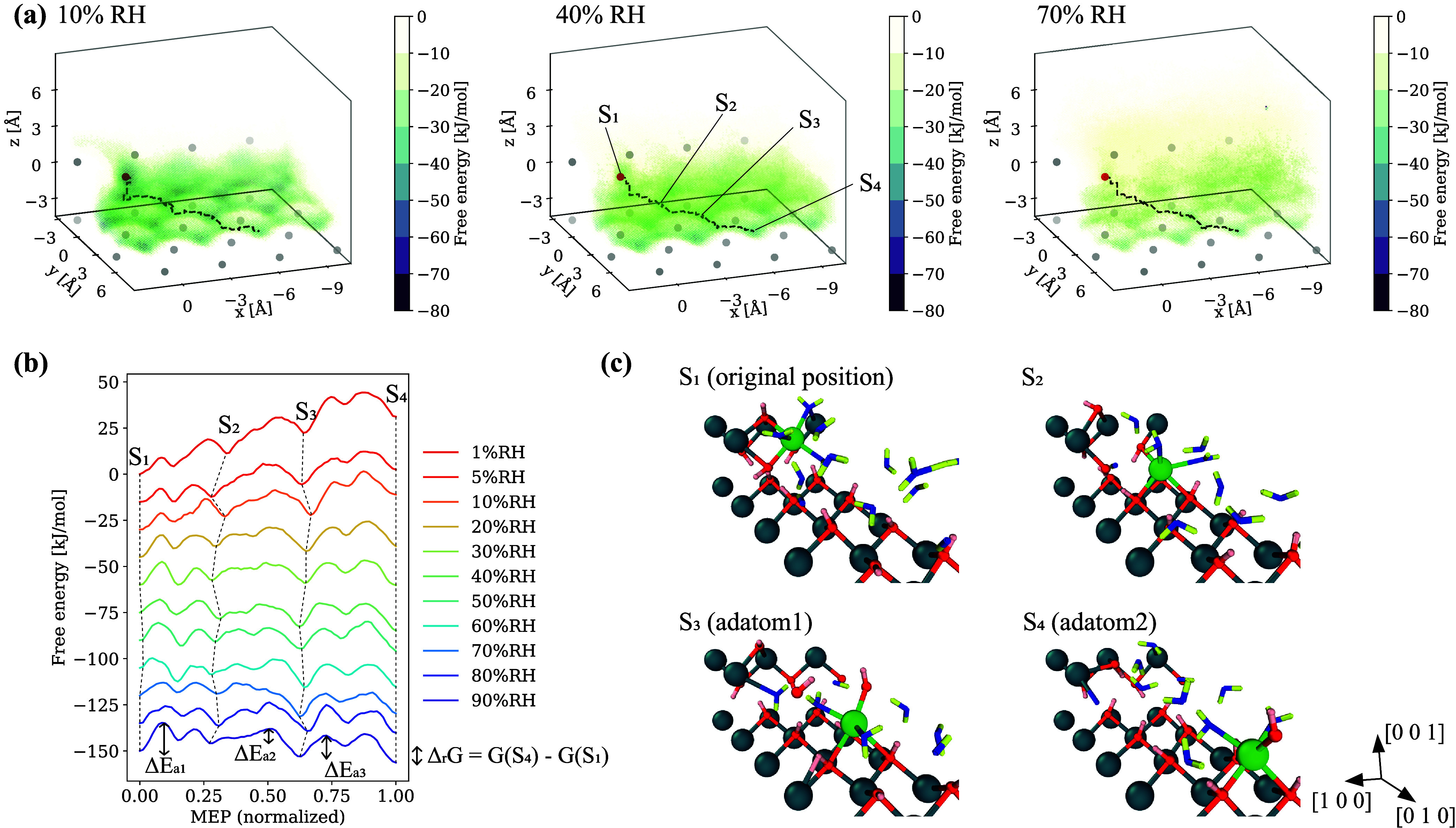
(a) Free energy surfaces of the kink Ca (marked red) on the (0 0 1) surface at 10, 40, and 70% RH. *x*, *y*, and *z* axes show the relative displacement of Ca from the original position. The minimum energy path (MEP) from the kink to an adatom is overlaid in a dotted line. (b) Extracted free energies along MEP at various RH. Each line is offset by 15 kJ/mol for visualization. (c) Snapshots at some stable positions. The colors of atoms are the same as Figure 4 with the Ca of interest marked green.

The results of the (0 1 0) kink site are shown in Figure 10. Similar to (0 0 1), there were characteristic FES patterns that represented low-energy sites, but the patterns were rectangular in shape. These rectangles were parallel to (0 1 0) surfaces and above the underlying Ca layer. The local minimum energy sites were located at the vertices of the rectangles, where the dissolved Ca was bonded to three Oh or Ow that also bonded to Ca in the substrate layer (see the snapshots of S_3_ and S_4_). These sites can also be regarded as IS. Figure 11 summarizes the results of the step and terrace sites on (0 0 1). FES shows that Ca was likely to be trapped on hexagonal patterns parallel to the surface, staying at the local minima similar to those of kink.

**Figure 10:**
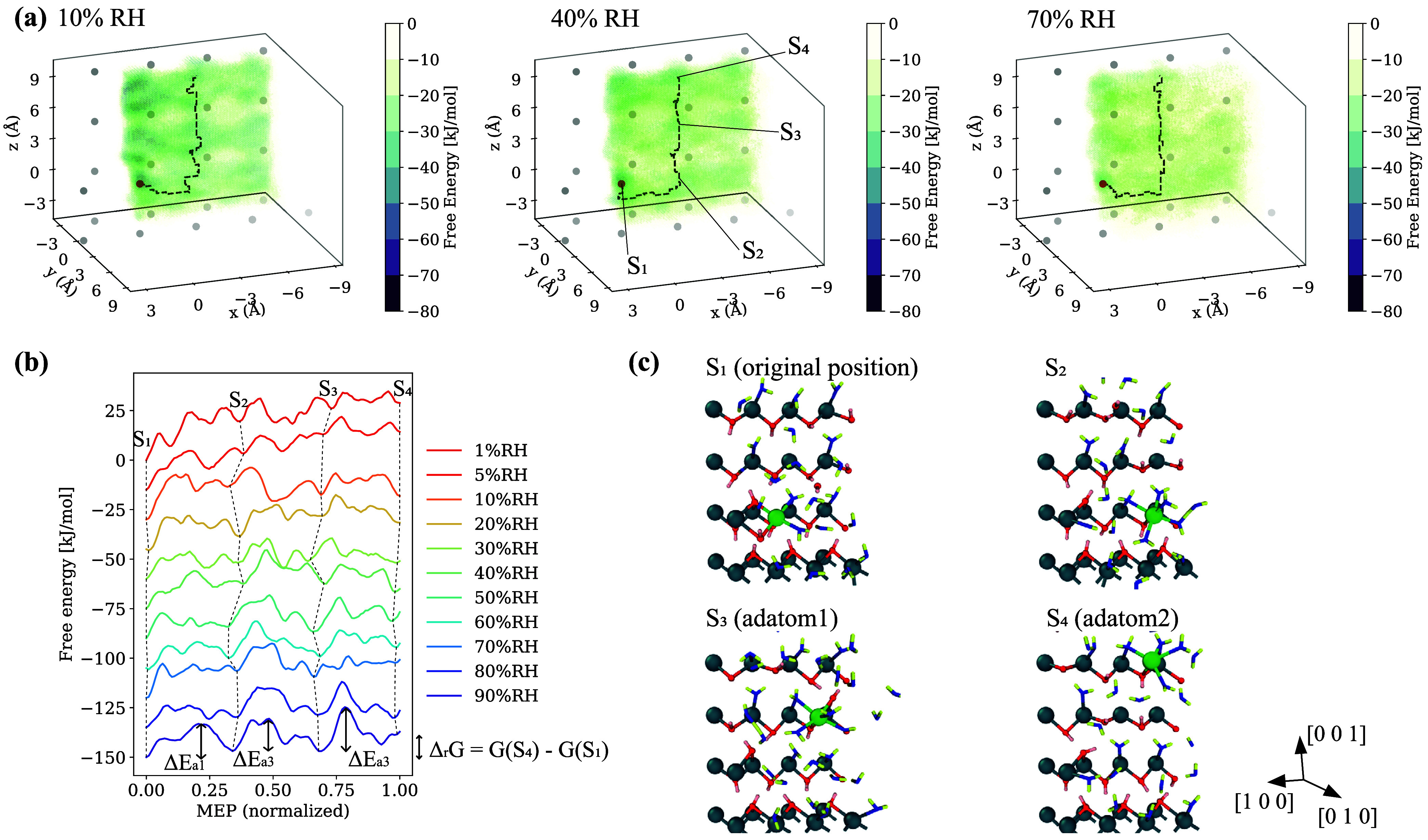
(a) Free energy surfaces of the kink Ca (marked red) on (0 1 0) surface at 10, 40, and 70% RH. *x*, *y*, and *z* axes show the relative displacement of Ca from the original position. The minimum energy path (MEP) from the kink to an adatom is overlaid in a dotted line. (b) Extracted free energies along MEP at various RH. Each line is offset by 15 kJ/mol for visualization. (c) Snapshots at some stable positions. The colors of atoms are the same as Figure 4 with the Ca of interest marked green.

**Figure 11:**
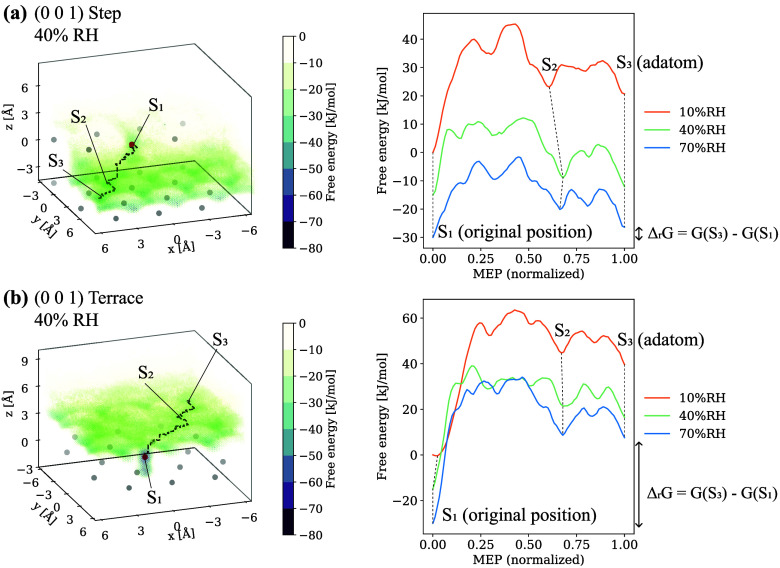
(a) Free energy surface of Ca for the (0 0 1) step at 40% RH (left) with the MEP overlaid and extracted free energies along MEP at various RH (right). (b) Same as (a) for the (0 0 1) terrace.

The activation energies between the local minima, Δ*E*_a1_ to Δ*E*_a3_, are summarized in Table 2. It is noted that the Gaussian height of the metadynamics simulation (=1.3 kJ/mol) and the thermal fluctuation of molecular dynamics (*k*_B_ *T*, ∼2.4 kJ/mol at 293.15 K) limited the precision of the values. Δ*E*_a1_ indicates the energy required for the Ca atom at kink/step/terrace to detach from its neighboring Ca and Oh atoms. Compared on the (0 0 1) surface at the same RH, Δ*E*_a1_ ascended in the order of kink, step, and terrace. This was intuitive because the number of neighboring atoms that form bonds with the dissolving atom had a strong effect on FES,^54,56^ with a larger number of neighbors increasing the energy barrier. The terrace site on (0 0 1) was surrounded by 6 neighboring Ca and Oh, while the step site (4 Ca and 5 Oh) and the kink site (3 Ca and 4 Oh) were less attracted by their neighbors. When comparing Δ*E*_a1_ among different RH, a decreasing trend can be found as the RH increased. As snapshots in Figures 9 and 10 show, the dissolved Ca formed bonds with surrounding Ow atoms on its way. This Ca–Ow bond played a role in decreasing free energy, but the lack of H_2_O at low RH did not result in that, which caused an increase in the energy barrier.

**Table 2:** Summary of the Activation Energy in kJ/mol for the (0 0 1) Kink and the (0 1 0) Kink

	RH (%)										
	1	5	10	20	30	40	50	60	70	80	90
(0 0 1)_kink_ Δ*E*_a1_	20.69	10.71	19.52	14.07	13.81	8.56	10.82	7.87	8.29	12.24	16.91
(0 0 1)_kink_ Δ*E*_a2_	21.00	19.68	16.17	12.76	10.98	8.78	8.67	9.67	7.95	14.91	9.14
(0 0 1)_kink_ Δ*E*_a3_	23.49	19.06	24.57	17.70	14.40	14.36	17.34	13.69	16.76	15.89	13.93
(0 0 1)_step_ Δ*E*_a1_			47.39			28.37			29.96		
(0 0 1)_step_ Δ*E*_a2_			7.96			12.54			10.11		
(0 0 1)_terrace_ Δ*E*_a1_			65.05			57.05			66.07		
(0 0 1)_terrace_ Δ*E*_a2_			12.15			11.00			13.58		
(0 1 0)_kink_ Δ*E*_a1_	29.22	22.04	24.50	20.81	15.17	20.35	13.90	15.14	19.03	13.01	19.69
(0 1 0)_kink_ Δ*E*_a2_	12.82	11.71	11.03	17.14	13.16	18.63	17.04	19.93	14.64	15.75	17.43
(0 1 0)_kink_ Δ*E*_a3_	10.18	9.40	12.29	12.50	13.20	10.29	18.72	14.54	11.75	18.95	23.70

Δ*E*_a2_ and Δ*E*_a3_ are the energy required for an adatom to move toward another adatom. This surface diffusion process^39^ was described as a crawling behavior on the surface in a previous study.^110^ The values of Δ*E*_a2_ did not differ among different surface sites, suggesting that once the atom detached from its original positions, the subsequent energy barrier for its lateral movement was not significantly different.

The activation energy of CH dissolution in bulk H_2_O has been experimentally studied and reported to be 13.7,^111^ 13.9,^112^ 15.2,^113^ or 29.7 ^114^ kJ/mol. Our Δ*E* values at high RH (i.e., closer to bulk H_2_O) were consistent with these values. It is noted that the rate-limiting step for dissolution is considered a surface-controlled process rather than a transport-controlled process in the bulk solution, which corresponds to detachment from the kink site (corresponding to Δ*E*_a1_), surface diffusion (Δ*E*_a2_ and Δ*E*_a3_), or dissolution into bulk (described later, with the energy barrier being <20 kJ/mol at high RH).^115^ Similar activation energies among these elementary reactions in our results indicate that either reaction can be the rate-limiting step. Relatively lower activation energy of CH dissolution than other inorganic minerals (e.g., 35.1 kJ/mol for calcite^116^ and 41.8 kJ/mol for gypsum^117^) makes the identification of the rate-limiting step difficult.

Another key insight revealed in Figures 9 to 11 is the difference in free energy (Δ_*r*_ *G*) between the original site and the adatom, because it can be used to judge whether the reaction is spontaneous (exergonic) or not (endergonic). Δ_*r*_ *G* is plotted in Figure 12. Focusing on the (0 0 1) kink site, a bilinear trend was observed: at 40% RH or below, Δ_*r*_ *G* linearly decreased with increasing RH. It first took a negative value at 40% RH and a further increase in RH than 40% did not make a large change of Δ_*r*_ *G*, always taking negative and stable values independent of RH. The positive Δ_*r*_ *G* at 30% RH or below implied that the movement from the kink to the adatom was not thermodynamically favorable. This conclusion is well consistent with the experimental results in Figure 8, which showed that carbonation rarely occurred under 30% RH, as well as previous experiments.^30–32^

**Figure 12:**
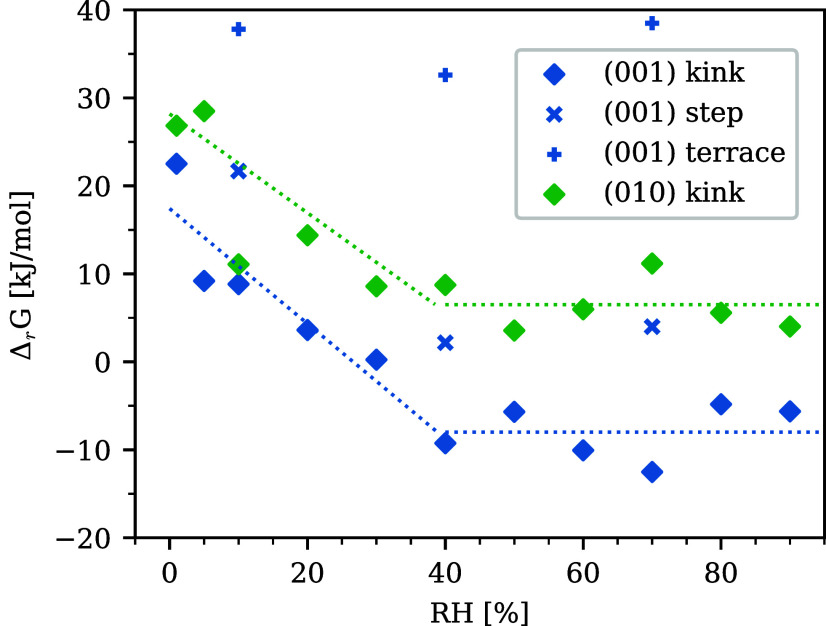
Δ_*r*_ *G* between the surface sites (kink, step, and terrace) and the adatom site. Dotted lines are the regressions for the (0 0 1) and (0 1 0) kinks.

The bilinear trend for Δ_*r*_ *G* was also found for the (0 0 1) step, although the absolute values of Δ_*r*_ *G* were shifted higher than those for the kink. As in the case of Δ*E*_a1_, a larger number of Ca–Oh bonds at the initial configuration made the original site energetically more stable than the kink. The (0 0 1) terrace was more strongly bound to the initial position, so Δ_*r*_ *G* was even higher. The finding that the kink site was the most reactive was supported by earlier experimental observations^118,119^ showing that CH dissolution begins at the etch pits and spreads in a screw-like manner.^42^

For the (0 1 0) kink site, Δ_*r*_ *G* was all positive even at high RH, suggesting that the move from the original position to the adatom theoretically did not happen. This does not match an earlier observation of Ruiz-Agudo et al., who also observed the progress of dissolution from (0 1 0).^42^ Despite this discrepancy, the bilinear of Δ_*r*_ *G* divided at 40% RH was also observed in a manner similar to the (0 0 1) kink.

We also performed another metadynamics simulation on how the dissolved Ca could move into the bulk solution from the adatom using a one-dimensional CV perpendicular to the surface. The results on (0 0 1) surface are summarized in Figure 13. The density profile of H_2_O perpendicular to the surface, taken from Figure 4 was also overplotted for discussion. The free energy was offset so that the position of the adatom was set to 0 kJ/mol for all RH.

**Figure 13:**
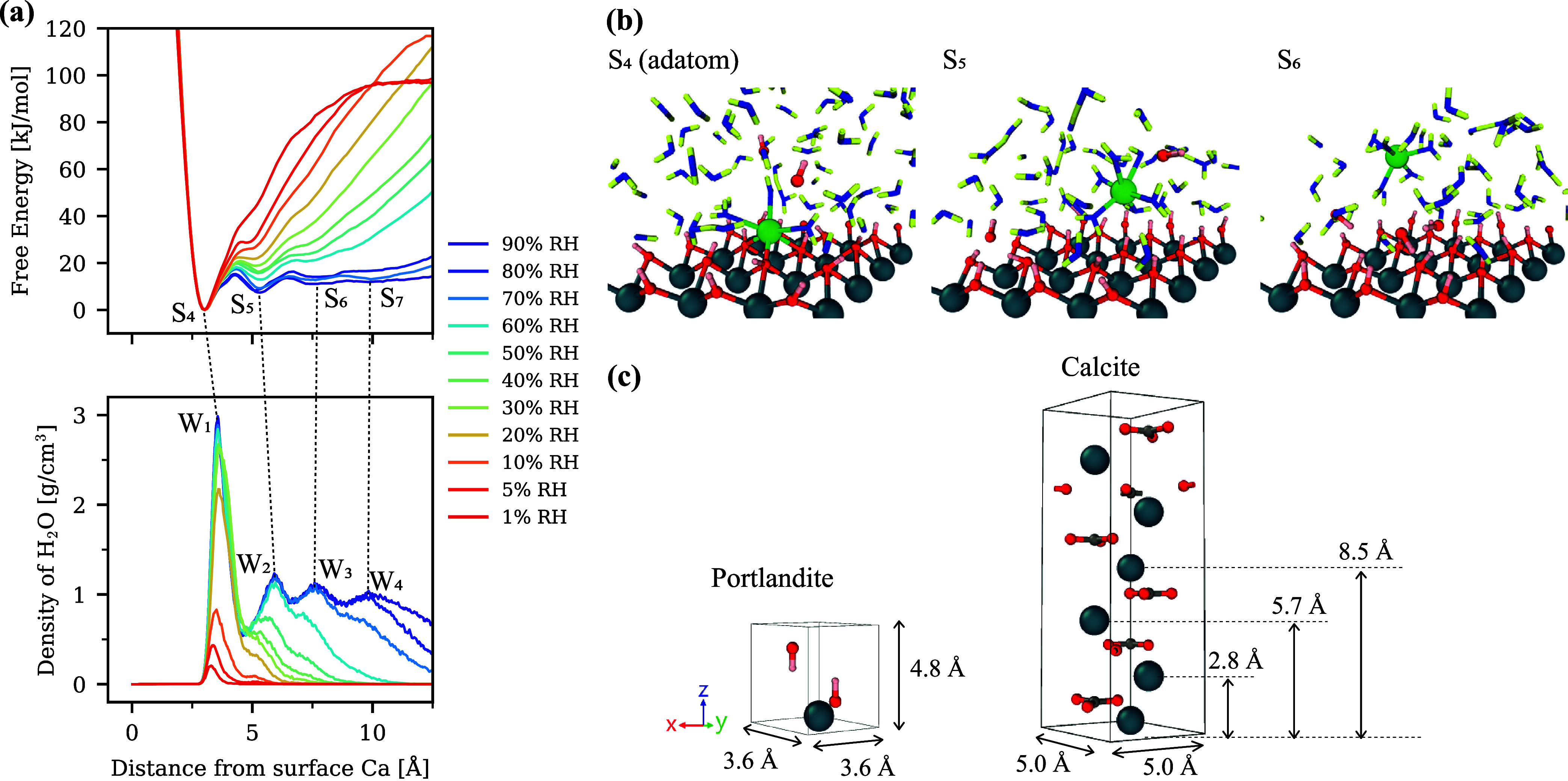
(a) Free energy surfaces of the dissolved Ca as a function of distance from (0 0 1) surface (top) and density profiles of H_2_O perpendicular to the (0 0 1) surface (below, taken from Figure 4). The underlying Ca layer was set to 0 Å. (b) Snapshots at stable configurations, taken from the simulation at 60% RH. (c) The unit cells of portlandite and calcite.

Figure 13a reveals that the energy barrier as well as the energy difference between the adatom (S_4_) and the stable configurations in the bulk (S_5_ to S_7_) were higher at lower RH, suggesting that the dissolution into the bulk was less likely. H_2_O density profile shows that each stable configuration of Ca was located near the H_2_O peaks, which means that its movement was strongly restricted by the presence of water. It is well-known that Ca cation in the solution hydrates with H_2_O, the coordination number being six to ten.^105,120,121^ The dissolved Ca in the snapshots of Figure 13b formed a 6-fold coordination with Oh or Ow atoms. When it was at S_4_ (adatom), there were three bonds with the surface Oh, two with Ow in the H_2_O monolayer (W_1_), and one with Ow in the second layer (W_2_). When it was at S_5_ (first stable position after leaving the adatom, located at ∼5 Å away from the underlying Ca layer), chemical bonds with Ow in W_1_, W_2_, and W_3_ were observed, the total coordination number being six. Similarly, at S_*n*_ positions (*n* = 6, 7, ...), Ca formed bonds with W_*n*–4_, W_*n*–3_, and W_*n*–2_ H_2_O layers.

However, this was only the case where there was sufficient amount of water: Ca in S_*n*_ could be 6-fold coordinated only when H_2_O layer was present not only at W_*n*–4_ or W_*n*–3_ but also at W_*n*–2_. A typical example where this condition was not met is the snapshot at S_6_ in Figure 13b. Since there were not enough Ow atoms in W_4_ at 60% RH, the dissolved Ca could form bonds with W_2_ and W_3_ but not with W_4_, which resulted in the total coordination number of five. The decrease in coordination number led to an increase in free energy by ∼15 kJ/mol at S_6_ position compared to the higher RH where Ca could connect six Ow atoms.

At 70% RH or higher, the dissolved Ca could move from S_4_ to S_7_ or further with energy barriers less than 20 kJ/mol. This high mobility of Ca could have helped to enhance subsequent ion transport and Cc precipitation. Previous studies demonstrated that the precipitation process involves (i) the formation of prenucleation clusters (PNC) from Ca^2+^ and CO_3_^2–^ ions,^108^ (ii) the agglomeration of clusters, and (iii) the transformation of agglomerated amorphous calcium carbonate (ACC) into more stable Cc phases through dissolution/reprecipitation.^107^ The minimum structural motif of the initially formed PNC is suggested to be 6 to 11 s Å by cryo-TEM experiments^122^ or in several pairs of ions by MD calculations.^123^ This size corresponds to the water film thickness of 70 to 80% RH, suggesting that the nucleation of PNC in bulk H_2_O can be possible at such high RH. In addition, Ruiz-Agudo et al. observed the epitaxial growth of calcite on portlandite,^42^ sharing the (0 0 1) plane in the way described in Figure 13c. The atomic configuration of calcite repeats Ca every 2.8 Å perpendicular to (0 0 1). The more dissolved Ca can go away from the surface, the more volume is geometrically available for Cc precipitation. As Figure 13a shows, a decrease in RH caused a decrease in H_2_O layers and Ca mobility, which spatially limited the possible volume for Cc nucleation and crystal growth. Consequently, the precipitation was likely to occur near the portlandite–aqueous interface.^31^ The Cc layer formed at these RH has been reported to be less permeable and inhibit further carbonation,^43^ which we consider the cause of the stoppage of carbonation observed in the experiments (Figure 8).

### Summary of the Relationship between Adsorbed Water and Carbonation Reactivity

Based on the experimental adsorption isotherm (Figure 2) and DoC (Figure 8), the relationship between the final DoC values and the statistical water film thickness (*n*_H_2_O_) is plotted in Figure 14. *n*_H_2_O_ is the number of H_2_O layers relative to the monolayer adsorption from the BET analysis (*v*_m_ = 0.800 cm^3^(STP)/g, see Supporting Information).

**Figure 14:**
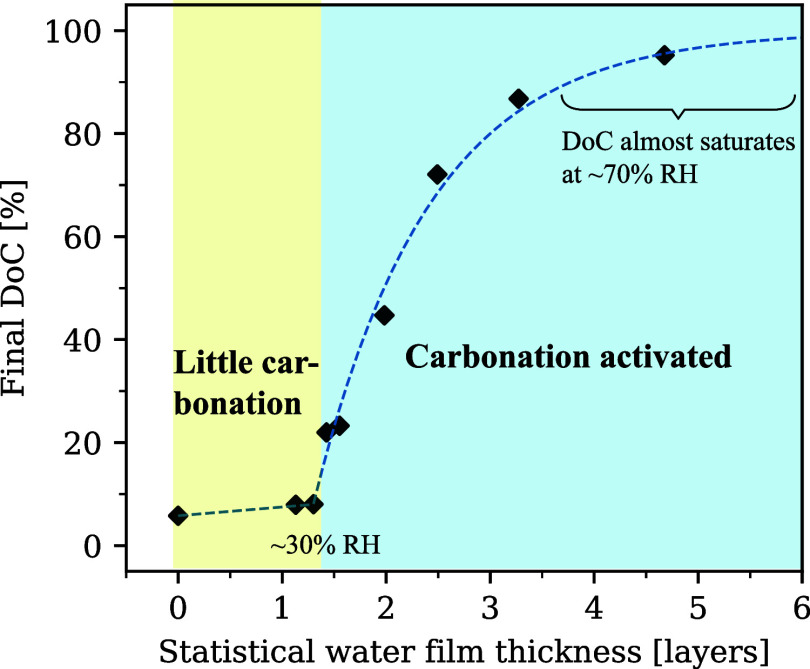
Final degree of carbonation (eq 5) as a function of the statistical water film thickness. The explanation of each zone is described in the text. The dotted curve at *n*_H_2_O_ > 1.3 is fitted by an exponential function in eq 9.

When *n*_H_2_O_ was below 1.3, carbonation rarely occurred. This corresponded to RH below ∼30%. The inertia in this zone can be explained by the simulations of Δ_*r*_ *G* for the detachment from the surface sites (Figure 12), which suggested that the dissolution of Ca was not thermodynamically favorable at such low RH. Above that *n*_H_2_O_, carbonation was enabled and the final DoC monotonically increased with increasing *n*_H_2_O_. It asymptotically approached 100% and could be well fitted with an exponential curve in eq 9.

9 $$\mathrm{DoC}=1-\left(1-0.08\right)e^{-a\left(n_{H_{2}O}-1.3\right)}$$

where 1.3 was the threshold *n*_H_2_O_ layers where carbonation activated and 0.08 was the corresponding DoC, both of which were introduced to offset the starting point of the exponential curve. *a* was the parameter fitted by the least-square method, which yielded *a* = 0.90. Some previous studies used a sigmoidal function for the fitting,^30,124^ but we consider that the offset exponential describes the relationship better because carbonation reactivity was almost zero below the threshold. Interestingly, an exponential fitting has been also used to express the decrease in chemical potential of H_2_O as a function of *n*_H_2_O_ ^125,126^ (see Supporting Information for the detailed discussion). The relationship between chemical potential of adsorbed water and carbonation reactivity can be worth investigating in the future study.

The increase in DoC with water film thickness was consistent with the simulation results in Figure 13, which showed that dissolved Ca could move further from the surface at higher RH and that larger volume was available for the subsequent Cc precipitation. In particular, DoC almost reached a plateau (>90%) at ∼70% RH. This result matches Figure 13: at 70% RH or above, FES lay on a similar curve and further increase in RH did not change the FES. The corresponding *n*_H_2_O_ needed for this continuous progress of carbonation was ∼4. This number was previously suggested by experiments of Beruto and Botter.^41^ It is noted that it is also possible that the H_2_O produced during the reaction played a catalytic effect and promoted the reaction.^43^ Overall, the underlying mechanism behind the relationship between DoC and water film thickness in Figure 14 can be well explained by the free energy simulations.

## Conclusion

The adsorption of H_2_O on portlandite and its effect on carbonation reactivity were investigated. GCMC-MD simulations revealed that the adsorption isotherms, the density profiles and the self-diffusion coefficient of the adsorbed H_2_O were surface dependent between (0 0 1) and (0 1 0), which was attributed by the difference in the coordination number of Ca at the surface layer. With the adsorbed water film at various RH, biased MD simulations were performed using a well-tempered scheme to investigate how Ca dissolved from portlandite surface. The results revealed that the dissolved Ca was likely to move on the hexagonal or rectangle patterns parallel to the surfaces for (0 0 1) and (0 1 0), respectively, and it was adsorbed in the inner sphere (adatom). The energy barrier for the Ca atom to detach from its original site was higher at low RH, while the barrier for movement between adjacent adatoms did not depend on RH. The free energy difference between the original site and the adatom, Δ_*r*_ *G*, showed a bilinear relationship with RH where it decreased with increasing RH under 40% RH and was constant above that. The positive values of Δ_*r*_ *G* under 40% RH could imply that Ca dissolution was not spontaneous. Another free energy studies revealed that the perpendicular movement of the dissolved Ca was restricted within where the H_2_O layer was present, which may also spatially limit the subsequent nucleation and crystal growth of calcium carbonate and inhibit the complete carbonation of the substrate. Furthermore, experiments were also performed to study the degree of carbonation (DoC) of portlandite at various RH. The experimental trend showed good consistency with simulations with respect to the reactivity threshold and the reaction-saturated RH.

As noted earlier, our simulations are simplified in two respects. First, we employed ClayFF with rigid H_2_O and OH^–^ molecules for dissolution, that is, chemical reactions which potentially involve bond breaking and recreation of proton-containing molecules. In this sense, the obtained Δ*E* and Δ*G* values are inevitably model-dependent, although the simulations in ReaxFF yields close FES to those obtained in ClayFF (Supporting Information). Second, the pore solution of cementitious systems contains not only Ca–O–H but also other ion species as well as carbonate species when it comes to carbonation, which might affect dissolution.^99,100^ However, it is necessary to first understand the CH dissolution in the basic setups before tackling the case with such a multicomponent solution. In this regard, our work provided a strategy that can be extended to these scenarios with more complicated alkali solutions. The methodology used here is ubiquitous and can be applied to other minerals relevant for cementitious systems, which has been gaining more and more attention as a way to mitigate CO_2_ emission.

## Supplementary Material



## Footnotes

1 In cement chemistry, C stands for CaO, S = SiO_2_, H = H_2_O, c = CO_2_.

## Notes

Force field for simulations, the evolution of the number of H_2_O during GCMC simulations, raw isotherm data and BET analysis, monitored RH for each carbonation desiccator during experiments, free energy surfaces at various RH not shown in the main text, free energy surface using ReaxFF, and discussion on chemical potential of adsorbed H_2_O (PDF)
