# Supplementary material for: Effects of Relative Humidity on Dissolution and Carbonation Potential of Portlandite
Source: Langmuir. 2026 Feb 23;42(9):6745–60. doi: 10.1021/acs.langmuir.5c05760 (PMC12980848; doi:10.1021/acs.langmuir.5c05760)
Supplement: Supplementary file 1 [file la5c05760_si_001.pdf]

Supporting Information: Effects of relative humidity on dissolution and carbonation potential of portlandite

Naohiko Saeki,*,† Ippei Maruyama,† and Tulio Honorio‡

†*Department of Architecture, Graduate School of Engineering, The University of Tokyo, 113-8656, Tokyo, Japan*

‡*Université Paris-Saclay, CEA, Service de recherche en Corrosion et Comportement des Matériaux, 91191, Gif-sur-Yvette, France*

E-mail: n.saeki@bme.arch.t.u-tokyo.ac.jp

ClayFF parameters

Table S1: Two-body parameters of ClayFF.[S1]

Species	Symbol	charge	ϵ [kcal/mol]	σ [Å]
Hydroxide calcium	Cah	1.0500	5.0298E-6	5.5667
Hydroxyl hydrogen	Hh	0.4250	-	-
Hydroxyl oxygen	Oh	-0.9500	0.1554	3.1655
SPC/E Water hydrogen	Hw	0.4238	-	-
SPE/E Water oxygen	Ow	-0.8476	0.1554	3.1655

Table S2: Bond parameters of ClayFF[S1]

	Species i	Species j	k_1 [kcal/mol/Å^2]	r_0 [Å]
SPC/E Water	O*	H*	554.1349	1.0000

Table S3: Angle parameters of ClayFF[S1]

	Species i	Species j	Species k	k_2 [kcal/mol/rad^2]	θ_0 [deg]
SPC/E Water	H*	O*	H*	45.7696	109.47

Time evolution of the number of H_2O during GCMC simulations

Changes of the number of H_2O molecules during GCMC simulations were plotted in Figure S1. At 60% RH or below, the number of H_2O was stabilized after 10 million GCMC trials. However, at higher RH, the number kept increasing after that due to capillary condensation: as Figure 1 in the main text shows, we created a 5 nm gap between surfaces. Kelvin radius r_K [nm] is calculated by

$$r_K = \frac{-2\gamma V}{RT\ln(p/p_0)} \quad (1)$$

where γ is the surface tension of water (0.0728 [N/m] at 20 C°), V is the molar volume of water, R is the gas constant, T is the temperature and $\frac{p}{p_0}$ is the relative pressure. At 70%

RH at 20 C°, r_K is 3.02 nm. Suppose a cylindrical pore, the diameter of the pore d where condensation occurs is $d = 2r_K = 6.04$ nm.[S2] This is wider than our gap width. Therefore, the number of H_2O in the GCMC simulation at 70% RH or above continued to increase and did not stabilize after 10 million GCMC trials. However, simulated isotherms using 10 - 12 million GCMC trials yielded consistent curves (Figure 2 in the main text) with the experiments at 70% RH or above.

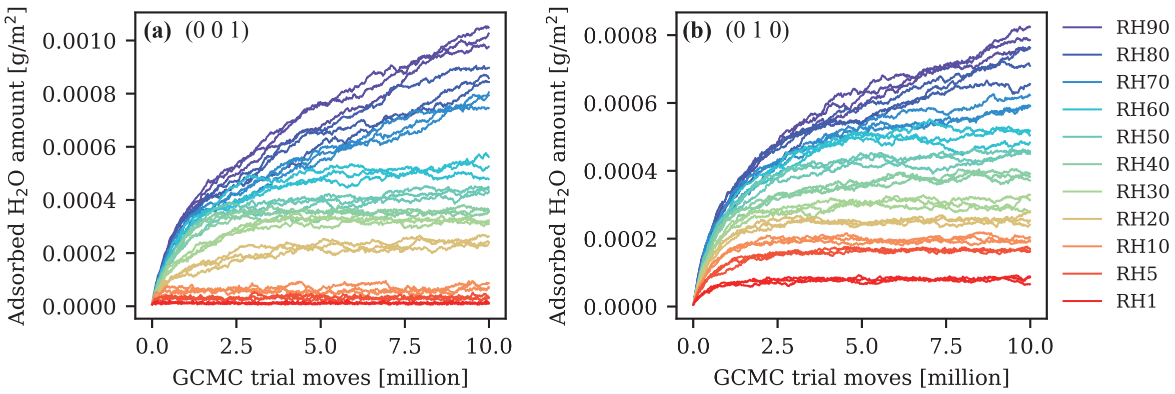

Figure S1: (a) Changes of the number of H_2O molecules during GCMC simulations for (0 0 1) and (b) (0 1 0). Three simulations were performed for each RH.

Raw experimental isotherm data

The raw isotherms are plotted in Figure S2. All three experiments can be plotted on an almost identical curve, which ensures repeatability. For Sample 1, BET analysis was performed within $0.05 < p/p_0 < 0.35$ range. The monolayer adsorbed H_2O amount (v_m) was calculated as 0.800 cm^3(STP)/g and the surface area was 2.34 m^2/g. The surface area was used in the main text to convert the adsorbed amount per surface area of CH. Also, v_m was used to calculate the statistical water film thickness.

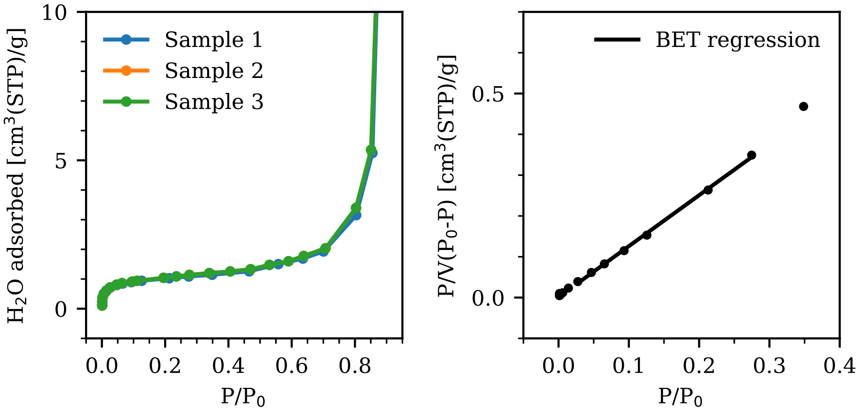

Figure S2: Experimental adsorption isotherms (left) and BET plot for sample 1 (right).

Measured RH during carbonation experiments

During carbonation experiments, RH was controlled with saturated salt solutions[S3] with KNO_3 (equilibrium RH at 20 °C is 95%), $(NH_4)_2SO_4$ (81%), NaCl(75%), KI (69%), NaBr (59%), K_2CO_3 (43%), $MgCl_2$ (33%), CH_3COOK (23%), and LiCl (11%). The RH in the desiccators during carbonation was monitored with data loggers (TR-76Ui, T&D) in every 15 minutes.

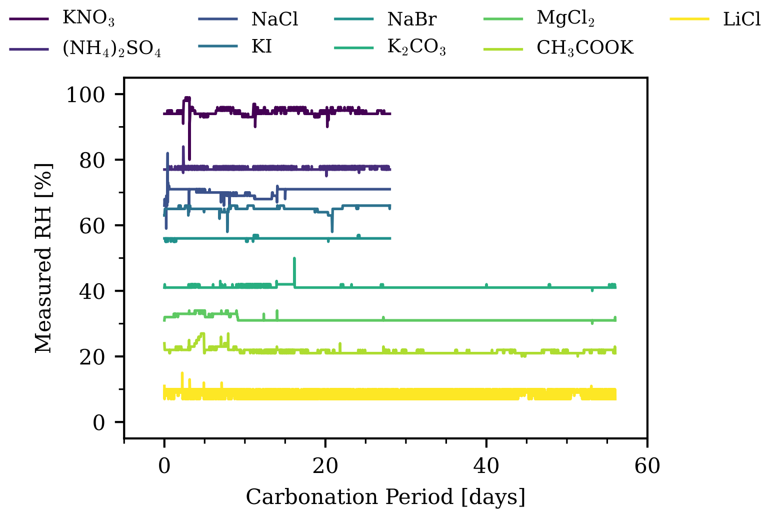

Figure S3: Measured RH during carbonation experiments.

Derivation of the solubility of CH

For the reaction simulated in this study in eq. (2), the reaction Gibbs free energy at T [K] is described in eq. 3.

$$Ca(OH)_2 \rightarrow Ca^{2+} + 2OH^- \tag{2}$$

$$\Delta_r G(T) = \Delta_r G^\circ(T) + RT \ln \frac{[Ca^{2+}][OH^-]^2}{[Ca(OH)_2]} \tag{3}$$

where $[Ca^{2+}]$, $[OH^-]$, and $[Ca(OH)_2]$ are the activities [mol/l], and $\Delta_r G^\circ(T)$ is the reaction Gibbs energy at standard state (1 mol/l) at T [K]. At T = 298.15 K,

$$\Delta_r G^\circ(298.15) = \Sigma_i v_i \Delta_f G^\circ(298.15) - \Sigma_j v_j \Delta_f G^\circ(298.15) \tag{4}$$

where v_i and v_j are stoichiometric values of reactants and products, and $\Delta_f G^\circ$ are their Gibbs standard energy of formation at 298.15 K.[S4] Calculating $\Delta_r G^\circ$ is +29.5 [kJ/mol-Ca(OH)$_2$]. The difference between 298.15 and 293.15 K is negligible. Substituting to eq. (3),

$$\Delta_r G(298.15) = +29.5 + 2.479\ \ln [Ca^{2+}][OH^-]^2 \tag{5}$$

Considering that the spontaneous reaction occurs only when $\Delta_r G < 0$, the requirement for CH to dissolve is

$$[Ca^{2+}][OH^-]^2 < e^{-11.9} (= 6.78 \times 10^{-6}) \tag{6}$$

Thus, reactivity depends on the activity of calcium and hydroxide ions. If there are only Ca^{2+} and OH^- ions in the solution, the activity of calcium divalent cation should be half of that of hydroxide anion to satisfy the overall electro-neutral condition. Solving eq. (6) under this condition, $[Ca^{2+}]$ is below 0.012 [mol/l] when $\Delta_r G < 0$.

Free energy surface with ReaxFF

As a preliminary test, we also performed the dissolution simulation from (0 0 1) kink site at 40% RH with ReaxFF. The ReaxFF parameters were cited from.[S5–S7] The timestep was 0.2 fs. The Gaussian potentials for each CV were 0.1 Å and the height was 0.3 kcal/mol, which were added every 100 timestep. We performed the simulation until 4 ns. In total, the number of the accumulated Gaussian bias (total of 200,000) was half of that in ClayFF simulations. The total simulation time (with MPI parallelization of 48 cores) took more than 1.5 months, which made ReaxFF simulations with three-dimensional CVs impractical.

The result is shown in Figure S4. Due to the shortage of simulation time, the area at x < -3 and y > 3 was not fully yet explored. However, a hexagonal pattern parallel to the (0 0 1) plane, which was observed in ClayFF simulation (See the main text), can be recognized. Compared to ClayFF simulations, the activation energies for kink dissolution (ΔE_{a1}) and between adjacent adatoms (ΔE_{a3}) were estimated higher.

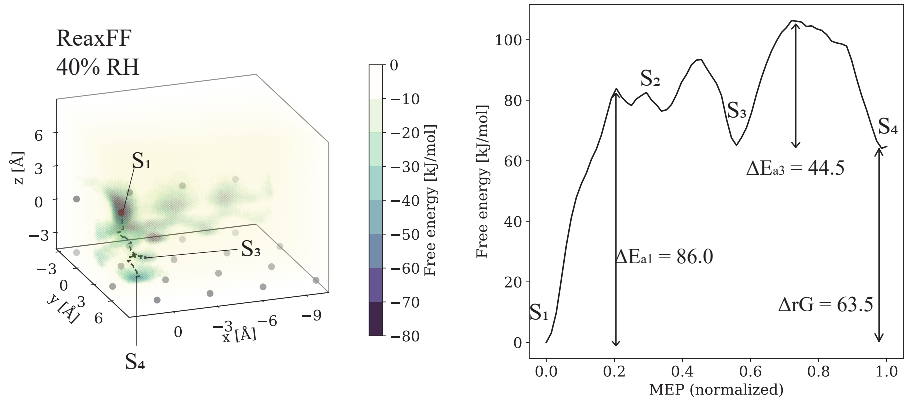

Figure S4: Free energy surfaces of the kink Ca (marked red) on (0 0 1) surface at 40% RH with ReaxFF parameters (left) and the extracted free energy along MEP (right).

Free energy surfaces at various RHs

Figure S5 and S6 show the FES of kink Ca at various RH, respectively. 10, 40, and 70% RH are summarized in the main text.

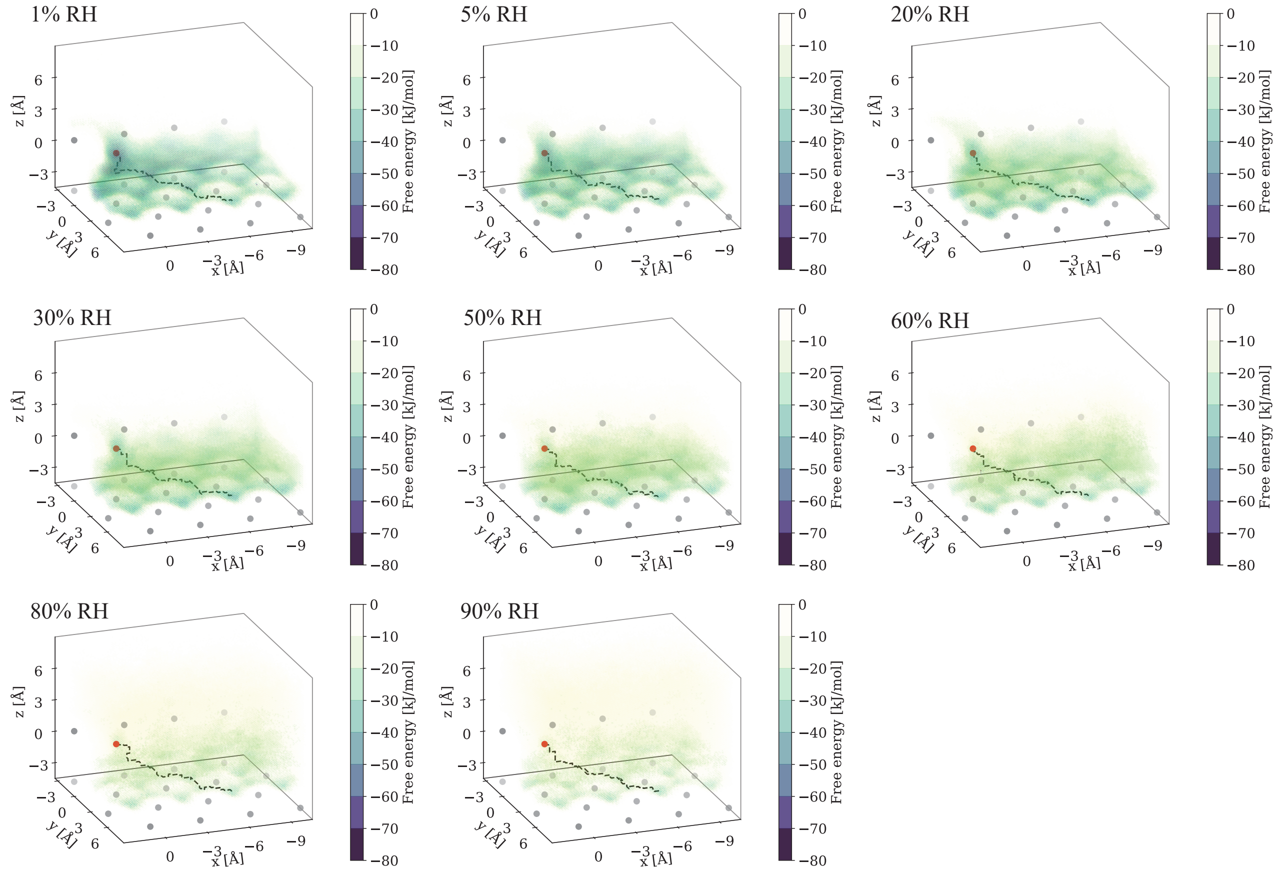

Figure S5: Free energy surfaces of the kink Ca (marked red) on (0 0 1) surface at various RH. X, y, and z-axis show the relative displacement of Ca from the original position. The minimum energy path (MEP) from the kink to an adatom is overlaid in a dotted line.

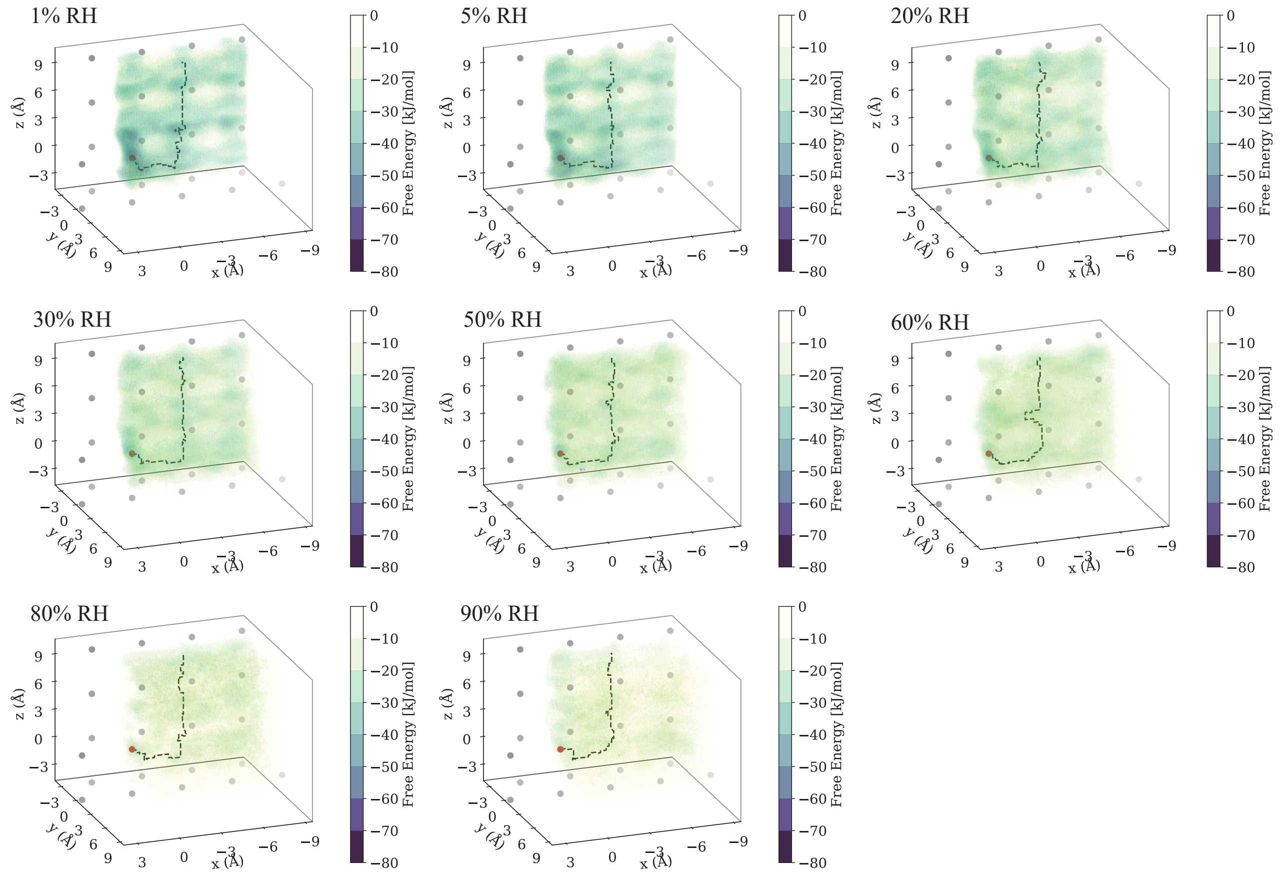

Figure S6: Same as Figure S5, for (0 1 0) kink Ca.

Chemical potential of the adsorbed H_2O

According to Badmann et al.,[S8] the statistical water film thickness n_{H_2O} (the unit was Å in their original article, but here we use [layers] in accordance with the main text) can be well fitted by a sigmoidal shape.

$$n_{H_2O} = K_1 + K_2(ln(-ln(RH))) \tag{7}$$

where K_1 and K_2 are constant and $0 \leq RH \leq 1$. $ln(RH)$ can be replaced by the decrease of chemical potential of H_2O from the reference state, $\Delta\mu_{H_2O}$, using the following equation.

$$\Delta\mu_{H_2O} = RTln(RH) \tag{8}$$

$\Delta\mu_{H_2O}$ takes a negative value. By substituting equation (7) by (8) and replacing the coefficients appropriately, one can get

$$\Delta\mu_{H_2O} = \Delta\mu^{\circ}_{H_2O}\ exp(-n_{H_2O}) \tag{9}$$

where $\Delta\mu^{\circ}_{H_2O}$ is the reference chemical potential, called onset potential. Adolphs and Setzer[S9] proposed an empirical relationship between $\Delta\mu^{\circ}_{H_2O}$ and C parameter in BET analysis as

$$\frac{|\Delta\mu^{\circ}_{H_2O}|}{RT} = 1.8 + 0.4(lnC)^2 \tag{10}$$

Using the measured C value of 353 from our BET analysis (in Figure S2), $\Delta\mu^{\circ}_{H_2O}$ can be estimated -15.8 kJ/mol. Substituting this to equation (9), the decrease of chemical potential of water as a function of the statistical water film thickness can be plotted in Figure S7. At the onset of carbonation ($n_{H_2O} = 1.3$), $\Delta\mu_{H_2O}$ decreases -4.3 kJ/mol compared to the saturated case ($n_{H_2O} = \infty$). A further decrease in n_{H_2O} also causes a further decrease in $\Delta\mu_{H_2O}$, which may be relevant to carbonation-inert at lower RH. Note that the order of $\Delta\mu_{H_2O}$ is similar to the energy barrier for Ca dissolution and surface diffusion is 10 to 20

kJ/mol (see the main text).

The fact that both DoC and chemical potential can be well fitted by exponential curves suggests a connection between the two, which is worth investigating in the future study.

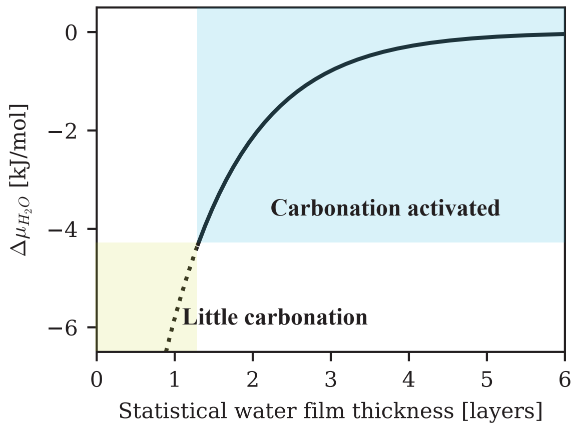

Figure S7: Decrease in chemical potential of H_2O ($\Delta\mu_{H_2O}$) as a function of the statistical water film thickness. The color separation is taken from Figure 14 in the main text, which separates the two zone at $n_{H_2O} = 1.3$.

References

(S1) Cygan, R. T.; Liang, J.-J.; Kalinichev, A. G. Molecular Models of Hydroxide, Oxyhydroxide, and Clay Phases and the Development of a General Force Field. *The Journal of Physical Chemistry B* **2004**, *108*, 1255–1266.

(S2) Aili, A.; Maruyama, I. Review of Several Experimental Methods for Characterization of Micro- and Nano-Scale Pores in Cement-Based Material. *International Journal of Concrete Structures and Materials* **2020**, *14*, 55.

(S3) Greenspan, L. Humidity Fixed Points of Binary Saturated Aqueous Solutions. *Journal of Research of the National Bureau of Standards Section A: Physics and Chemistry* **1977**, *81A*, 89.

(S4) Rumble, J. *CRC Handbook of Chemistry and Physics*, 106th ed.; Taylor & Francis Ltd, 2025.

(S5) Fogarty, J. C.; Aktulga, H. M.; Grama, A. Y.; Van Duin, A. C. T.; Pandit, S. A. A Reactive Molecular Dynamics Simulation of the Silica-Water Interface. *The Journal of Chemical Physics* **2010**, *132*, 174704.

(S6) Manzano, H.; Moeini, S.; Marinelli, F.; van Duin, A. C. T.; Ulm, F.-J.; Pellenq, R. J.-M. Confined Water Dissociation in Microporous Defective Silicates: Mechanism, Dipole Distribution, and Impact on Substrate Properties. *Journal of the American Chemical Society* **2012**, *134*, 2208–2215.

(S7) Manzano, H.; Pellenq, R. J. M.; Ulm, F.-J.; Buehler, M. J.; van Duin, A. C. T. Hydration of Calcium Oxide Surface Predicted by Reactive Force Field Molecular Dynamics. *Langmuir* **2012**, *28*, 4187–4197.

(S8) Badmann, R.; Stockhausen, N.; Setzer, M. J. The Statistical Thickness and the Chemi-

cal Potential of Adsorbed Water Films. *Journal of Colloid and Interface Science* **1981**, *82*.

(S9) Adolphs, J.; Setzer, M. J. A Model to Describe Adsorption Isotherms. *Journal of Colloid and Interface Science* **1996**, *180*, 70–76.
